# Combined Effects of Treatment and Sewer Connections to Reduce Future Microplastic Emissions in Rivers

**DOI:** 10.1021/acs.est.4c07730

**Published:** 2024-11-25

**Authors:** Tolga Ayeri, Yutong Guo, Peter J. T. M. van Puijenbroek, Nynke Hofstra, Ad M. J. Ragas, Maryna Strokal

**Affiliations:** † Department of Environmental Science, Radboud Institute for Biological and Environmental Sciences (RIBES), Radboud University Nijmegen, 6500 GL Nijmegen, The Netherlands; ‡ Aquatic Ecology and Water Quality Management Group, Wageningen University and Research, Droevendaalsesteeg 4, 6708 PB Wageningen, The Netherlands; § PBL Netherlands Environmental Assessment Agency, P.O. Box 30314, 2500 GH The Hague, The Netherlands; ∥ Earth Systems and Global Change Group, Wageningen University and Research, Droevendaalsesteeg 4, 6708 PB Wageningen, The Netherlands

**Keywords:** microplastics, modeling, wastewater treatment, reduction strategies, source-oriented, technology-oriented

## Abstract

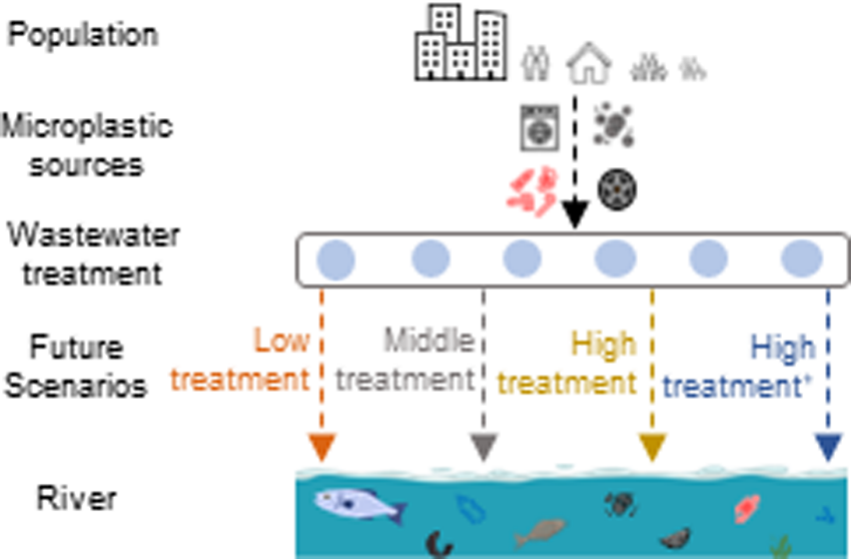

Global mitigation strategies are needed to reduce the amount of microplastics reaching our oceans via rivers. However, what strategies will be most effective, and when and where to implement these strategies is unclear. We applied the global water quality model MARINA-Plastics, covering 10,226 sub-basins worldwide, to assess the effects of different emission reduction strategies on microplastic inputs to rivers worldwide over the period 2010–2100, taking time steps of 10 years. We applied four scenarios: three focused on wastewater treatment technologies, ranging from high to low technology improvement levels, and one combining high technology in wastewater treatment with source-oriented measures. The results show that the combined strategy of high wastewater treatment and source-oriented measures is expected to be the most effective for reducing future microplastics in rivers on a global scale. By 2100, this combined strategy is expected to result in a 68% microplastic reduction in global rivers compared to 2010. African rivers will be the main hotspots, receiving more than five times more microplastics in 2100 than in 2010. In 2100, wear from car tires is expected to be the dominant source of microplastics globally. Our insights support the implementation of the European Green Deal and the realization of Sustainable Development Goal 6 (clean water).

## Abstract 2

This study compares the effectiveness of different strategies to reduce the emission of microplastics to rivers globally. It shows that a combined strategy of high technology in wastewater treatment and source-oriented measures will be most effective for reducing future microplastics in global rivers.

## 1 Introduction

Plastic pollution is one of the main global environmental challenges, particularly in aquatic environments such as oceans, rivers and streams.^1−4^ Approximately 10% of global plastic production ends up in the oceans.^5^ A significant part of this marine plastic pollution stems from land activities.^6^ Rivers and streams transport plastics from land to seas and oceans.^7−9^ Plastics can be degraded and fragmented in terrestrial and aquatic environments, ultimately ending up as microplastics (MPs) in our seas and oceans.^8^ Based on their origin, MPs are divided into two categories: primary and secondary MPs.^5,10,11^ Primary MPs are released directly into the environment, i.e., in the form of MPs.^5,8^ Secondary MPs are formed in the environment from the degradation and fragmentation of larger plastic items after exposure to sunlight, wind, water, and other environmental factors.^8,12^

MP pollution arises from point and diffuse sources.^6,13^ Sewer systems are typical point sources discharging MPs into rivers originating from laundry (i.e., textile fibers), personal care products (PCPs), household dust, and car tire wear.^1,8,14^ Surface runoff, rainfall and wind are examples of diffuse sources in aquatic environments.^6^ Diffuse sources are mainly responsible for macroplastics rather than MPs.^6,14^ However, the majority of MPs originate from sewer effluents, which are considered point sources.^6^ Therefore, this study focuses on sewer systems (i.e., point sources) in rivers.

Many global and regional models have been developed to simulate patterns in MP pollution.^1,6,8,15−17^ For instance, Schmidt et al. estimated global MP and macroplastic loads in river catchments based on mismanaged plastic waste.^15^ Siegfried et al. analyzed the transport of MPs from European rivers to the sea based on point source emissions; future trends in MP inputs (up to 2050) were also included.^1^ The Global Riverine Export of Microplastics into Seas (GREMiS) model was developed by van Wijnen et al. to quantify global MP export to coastal seas.^8^ Three different scenarios were simulated to assess future MP export by rivers. The global Model to Assess River INputs of pollutaNts to seAs (MARINA-Multi) was used to analyze the future trends in inputs of multiple pollutants in 10,226 river basins worldwide, including MPs.^18^ The model included five Shared Socioeconomic Pathways (SSPs) reflecting different levels of urbanization and wastewater treatment.^18^ Finally, the Model to Assess River Inputs of pollutaNts to seAs for plastics (MARINA-Plastics) was developed based on the MARINA-Multi model to quantify the river export of macro- and microplastics to seas from both point and diffuse sources worldwide.^6^

MP reduction strategies have not explicitly been considered in most MP modeling studies, except for a study with the MARINA-Multi model that focused on the Black Sea.^19^ This makes it difficult to assess whether current emission reduction strategies are in line with the Sustainable Development Goals (SDGs). Several approaches to reducing MP pollution exist such as end-of-pipe and pollution prevention.^20,21^ The end-of-pipe approach is widely used to control effluent discharges from wastewater treatment plants.^22,23^ It makes use of wastewater treatment technologies to reduce MPs before reaching the receiving environments, such as rivers. Besides the end-of-pipe approach, the pollution prevention approach can be used to reduce MP discharges into rivers.^24^ According to the United States Environmental Protection Agency, pollution prevention is defined as “the use of materials, processes or practices that reduce or eliminate the creation of pollutants or wastes at the source”.^25^ The pollution prevention approach can be implemented through legislation, action plans and behavioral change.^20,24,26^ In this research, we make use of the EU’s Zero Pollution Action Plan, which aims to reduce MP release into the environment by 30% in 2030.^27^

The main objective of the present study was to assess the effects of different emission reduction strategies on the MP inputs to rivers worldwide. To this end, the MARINA-Plastics model was applied to simulate future trends of MPs in rivers of 10,226 sub-basins over the period 2010–2100, taking time steps of 10 years. We applied three technology-oriented wastewater treatment scenarios, ranging from high to low technology improvement levels and one scenario combining high technology in wastewater treatment with source-oriented measures.^28^ Hotspots of MP pollution were identified in 2010, 2030, 2050, 2070, and 2100 for each of the four scenarios. Finally, dominant MP sources in these years were identified for one scenario as an illustrative example.

## 2 Materials and Methods

### 2.1 MARINA-Plastics Model

**Figure 1:**
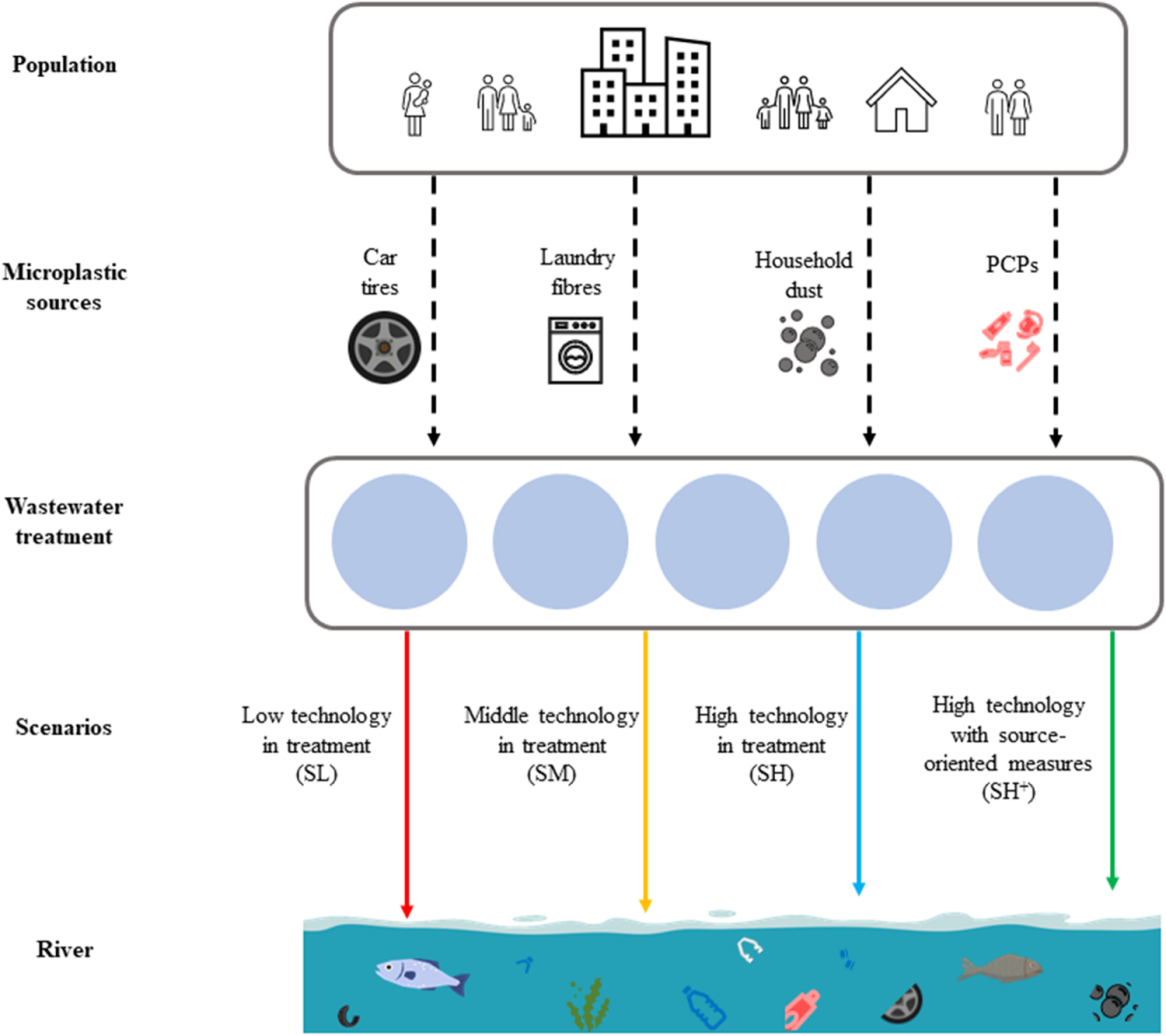
Schematic presentation of the MARINA-Plastics model. The model includes four microplastic sources and four scenarios (SL, SM, SH, and SH^+^) (PCPs = personal care products).

We used the MARINA-Plastics model to estimate MP inputs in rivers of 10,226 sub-basins from point sources between 2010 and 2100.^6^ The MARINA-Plastics model is capable of quantifying both point and diffuse sources in 10,226 sub-basins covering 187 countries (Table S1).^6^ However, we focus only on point sources, which come from sewer systems discharging MPs originating from laundry, car tires, household dust and personal care products. These inputs depend on the removal efficiencies during treatment.^18^ We also incorporated four scenarios into the model to explore future MP inputs in global rivers (see details below). The schematic presentation of the MARINA-Plastics model is shown in Figure 1.

The MARINA-Plastics model requires three main inputs: the population connected to the sewer system, consumption rates of MPs, and removal efficiencies of MPs during wastewater treatment (Tables S2–S4).^6^ First, sewer systems are described as the collection of wastewater from houses (MP from personal care products, household dust and laundry fibers) and streets (MP from car tires).^6^ The population numbers were derived from the global 0.125° cell database of Jones and O’Neill.^29^ We aggregated the population between 2010 and 2100 from 0.125° cells to 0.5° cells (Table S4). The fractions of urban and rural populations connected to sewer systems based on different scenarios were available from van Puijenbroek et al. by country over the period 2010–2100, taking time steps of 10 years.^28^ Second, consumption rates of PCPs, laundry fibers and household dust were directly derived from Siegfried et al.^1^ The approach for consumption rates of car tires presented by Siegfried et al. was modified by Strokal et al., distinguishing between developing countries (Human Development Index (HDI) < 0.785) and developed countries (HDI > 0.785) (details are in Strokal et al.).^30^ Finally, the removal efficiency of MPs during wastewater treatment was derived from the approach of Siegfried et al., relating average phosphorus removal in a watershed to MP removal (Table S4).^1^ Strokal et al. used the known phosphorus removal rate to assume the removal of MPs.^30^ The removal efficiencies do not differ between years. Further details about how model inputs are derived are presented in Supporting Tables S2–S4.

This paper follows the approach in the MARINA-Plastics model calculating river export of MP from the sewer system.^6^ This is calculated as follows
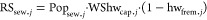
1where RS_sew.*j*_ is the MP input to rivers in sub-basin *j* from sewer systems (kg/year); Pop_sew.*j*_ is the population with sewer connection in sub-basin *j* (people/year); WShw_cap.*j*_ is the consumption rate of microplastics per capita in sub-basin *j* (kg/cap/year, Table S4); hw_frem.*j*_ is the removal fraction of microplastics during the wastewater treatment in sub-basin *j* (0–1, Table S4.e).

In this research, we enhanced the original MARINA-Plastics model by incorporating a new wastewater treatment level, called quaternary, which improved the calculation of the averaged removal fractions for MPs. While the original model included three treatment levels, our version adds this additional level to estimate future MP inputs to rivers from treatment facilities.^6^ In addition, we expanded the model’s application in two ways. First, we extended the model to estimate MP inputs into rivers over the 21st century (2010–2100) using 10-year time steps with our updated methodology for the treatment removals. Many existing studies on the MARINA models focused either only on 2050 and 2100,^18,19,31^ as two snapshot years for the 21st century or used the methodology for treatment that did not consider the quaternary treatment, which may underestimate the treatment efficiencies in some sub-basins.^19^ Second, four new scenarios based upon the Shared Socioeconomic Pathway 2 (SSP2) were integrated into the MARINA-Plastics model (see details below). These scenarios help to project future MP inputs in rivers worldwide (Table 1).

**Table 1:** Differences between the MARINA-Plastics Model and the Updated Version of the MARINA-Plastics Model (This Research)^a^

Difference	The current version of the MARINA-plastics^10,23^	The updated version of the MARINA-plastics model in this research	Explanations
Years	2010, 2050, and 2100	2010, 2020, 2030, 2040, 2050, 2060, 2070, 2080, 2090 and 2100	The MARINA-Plastics model focused on the current plastic export. This research adds the model for more years with updated methodology for the treatment removals: 2010, 2020, 2030, 2040, 2050, 2060, 2070, 2080, 2090 and 2100
Scenarios	SSP2-RCP2.6 as part of the multipollutant assessment	Four variations of SSP2: SL, SM, SH, SH^+^	The MARINA-Plastics model does not include four variations of SSP2 specifically for microplastics (SL, SM, SH, SH^+^)
Treatment levels	Primary, secondary, tertiary	Primary, secondary, tertiary and quaternary	The MARINA-Plastics model considers primary, secondary and tertiary wastewater treatment levels. This research adds the quaternary treatment as an additional treatment level^24^

a The SL, SM, SH, and SH^+^ represent the Shared Socioeconomic Pathway 2 low, moderate, high and high plus scenarios, respectively.

### 2.2 Scenarios

As a baseline scenario, this paper applies SSP2, which defines a narrative following the current social, economic and technological trends based on historical patterns.^32^ In the SSP2, known as the middle of the road scenario, global population growth and technological development are moderate.^32,33^ Socioeconomic challenges for mitigation and adaptation are intermediate.^32^ This study focuses on four variations of the SSP2: SSP2 low (SL), SSP2 moderate (SM), SSP2 high (SH) and SSP2 high plus (SH^+^) (Table 2). The development of socioeconomic parameters in these scenarios, such as population size, gross domestic product (GDP) and the human development index (HDI), is the same as in SSP2^34^ (Table 2).

**Table 2:**
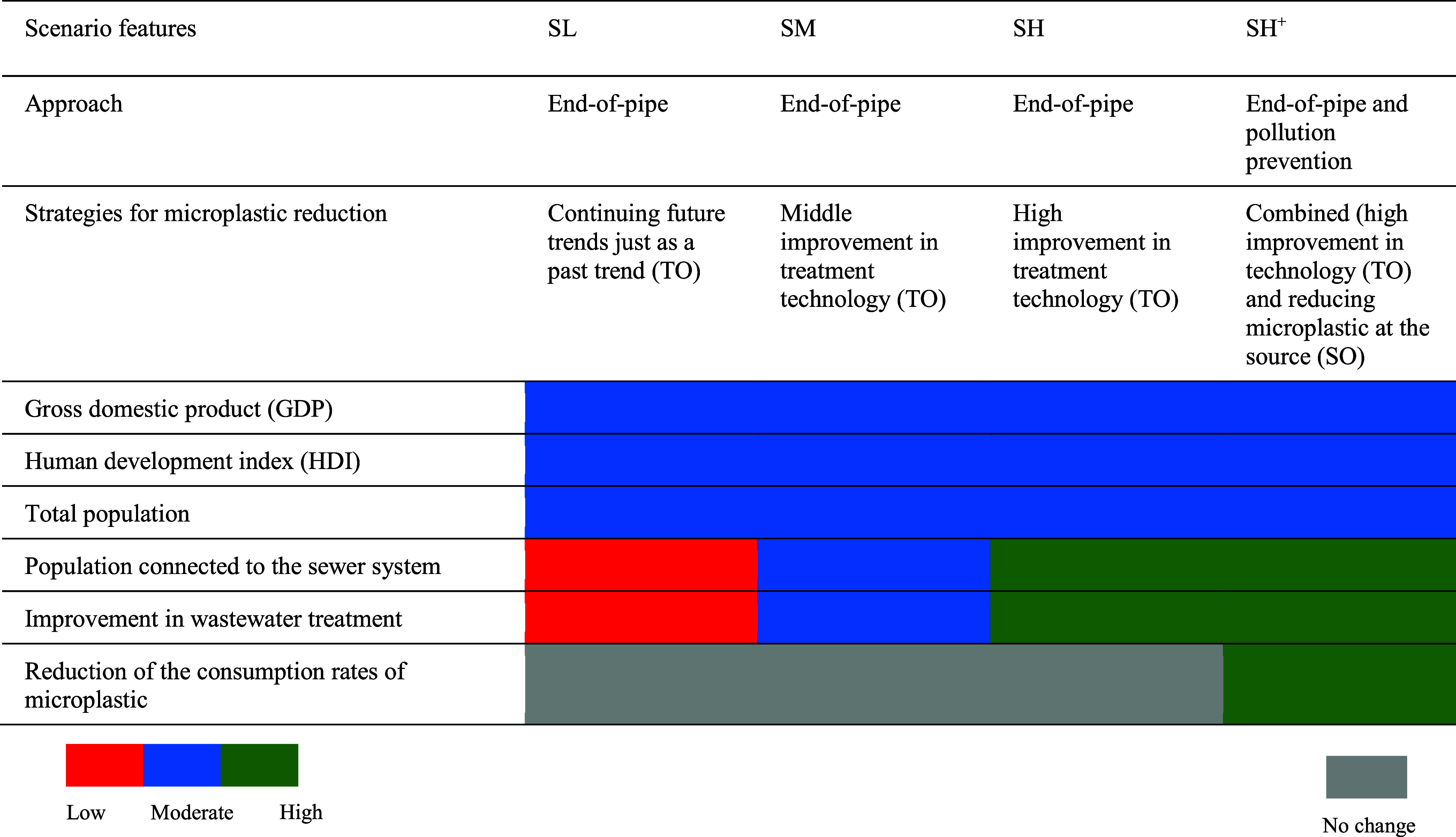
Scenario Descriptions and Features^a^

a Approaches and microplastic strategies are explained in the text. The SL, SM, SH, and SH^+^ represent the Shared Socioeconomic Pathway 2 low, moderate, high and high plus scenarios, respectively. TO and SO stand for technology-oriented and source-oriented strategies, respectively. Source: European Commission, Strokal et al., and van Puijenbroek et al.

Though the SL, SM, and SH scenarios follow the same end-of-pipe approach, the scenarios have different improvement levels for wastewater treatment (Table 2). The SL scenario has the lowest improvement level; it assumes the trend between 1970 and 2015 continues in the future. The SM scenario follows the SSP2 narrative, resulting in a middle improvement level. The SH scenario has the highest improvement level in wastewater treatment technologies.^28^ Finally, the SH^+^ scenario is the most optimistic, combining both the end-of-pipe and pollution prevention at the source approaches. The SH^+^ scenario, built upon SH, integrates a 30% reduction in MP at the source, aligning with the EU Zero Pollution Action Plan.^27^ The plan’s implementation assumes countries, categorized by HDI, will achieve a 30% MP reduction by specific future years, determined by HDI quartiles^35^ (Figure S1 and Table S4). For example, a country with an HDI between 0.55 and 0.62 is expected to reach the MP reduction target by 2040, maintaining a 30% reduction thereafter. In essence, we assume the consumption rate of MPs will decrease in the future in accordance with each country’s HDI. The selection of target years (2040, 2060, 2070) was arbitrary.

## 3 Results

### 3.1 Global Future Trends in Microplastic Inputs to Rivers

**Figure 2:**
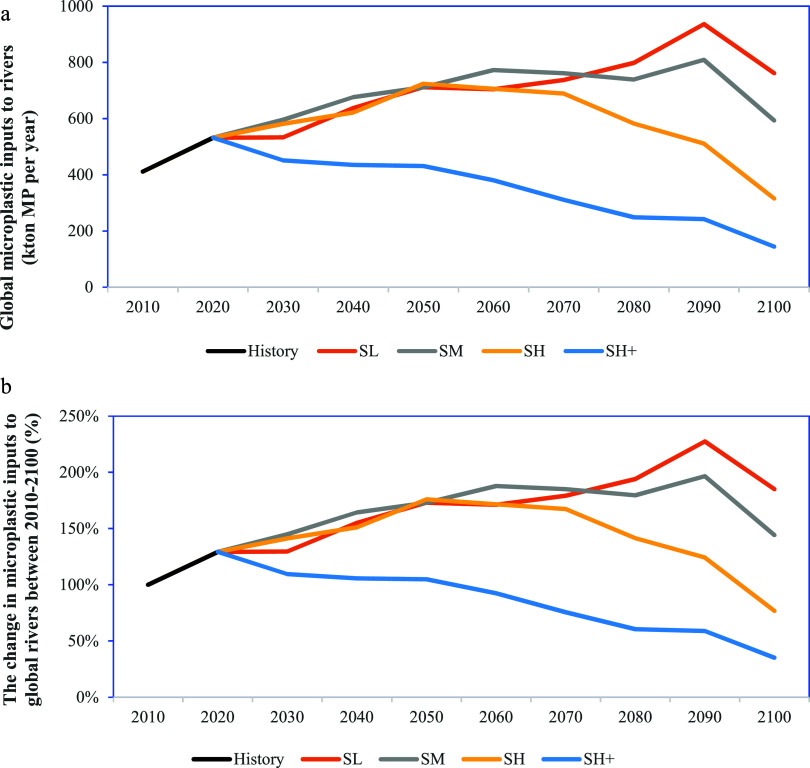
(a) Total microplastic inputs (kton microplastic year^–1^) to the world’s rivers in four scenarios over the period 2010–2100, time steps of 10 years. Each line represents a scenario. (b) The change in microplastic inputs to global rivers in four scenarios over the period 2010–2100, time steps of 10 years (%). The SL, SM, SH, and SH^+^ represent the Shared Socioeconomic Pathway 2 low, moderate, high and high plus scenarios, respectively.

Globally, over 400 kton of MPs were estimated to enter rivers from sewer systems in 2010 (Figure 2a). Between 2010 and 2100, MP inputs to rivers in the SH^+^ scenario are projected to be lower than those in the SL, SM, and SH scenarios, because of the higher removal efficiency and lower consumption rate of MPs in the SH^+^ scenario (Figure S2 and Table S4). This highlights the effectiveness of combining the high removal fractions during the treatment and source-oriented measures to reduce future MPs in global rivers (Figure 2).

In 2030, MP inputs into rivers in the SL, SM, and SH scenarios are projected to slightly increase compared to 2010 (Figure 2a). This trend is expected to continue until 2050, with an increase of more than 50% as the population with sewer connections is projected to increase by 2-fold in these scenarios (Figures 2b and S3). By 2070, MP inputs into rivers in SL, SM, and SH scenarios are expected to increase by more than 65% compared to 2010 because of an increase in the population connected to the sewer systems (Figures 2b and S3). Population growth with sewer connections is expected to outpace improvements in wastewater treatment efficiencies by 2070, leading to higher MP emissions into rivers in those scenarios For example, the population connected to sewer systems in the SL scenario is projected to more than double by 2070 compared to 2010, whereas removal fractions during wastewater treatment is expected to increase by only 22% (Figures S2 and S3).

In 2100, the combined strategy (the SH^+^ scenario) is projected to reduce MP inputs in global rivers by 68% compared to 2010 (Figure 2b) because of more countries reaching the 30% reduction target in the SH^+^ scenario. However, the SL and SM scenarios are projected to show an increasing trend of 85 and 44%, respectively (Figure 2b). From 2070 to 2090, MP in rivers globally is projected to continue increasing in the SL and SM scenarios (Figure 2a). This increasing trend is driven by the growing population connected to sewer systems (around 10% increase during 2070–2090, Figure S3). But this pollution trend is expected to shift from increase to decrease between 2090 and 2100 (Figure 2a). Improvements in wastewater treatment efficiencies are projected to outpace population growth with sewer connections between 2090 and 2100, causing a decrease in MP inputs into rivers in the four scenarios.

### 3.2 Hotspot Areas in Microplastic Inputs to Rivers

**Figure 3:**
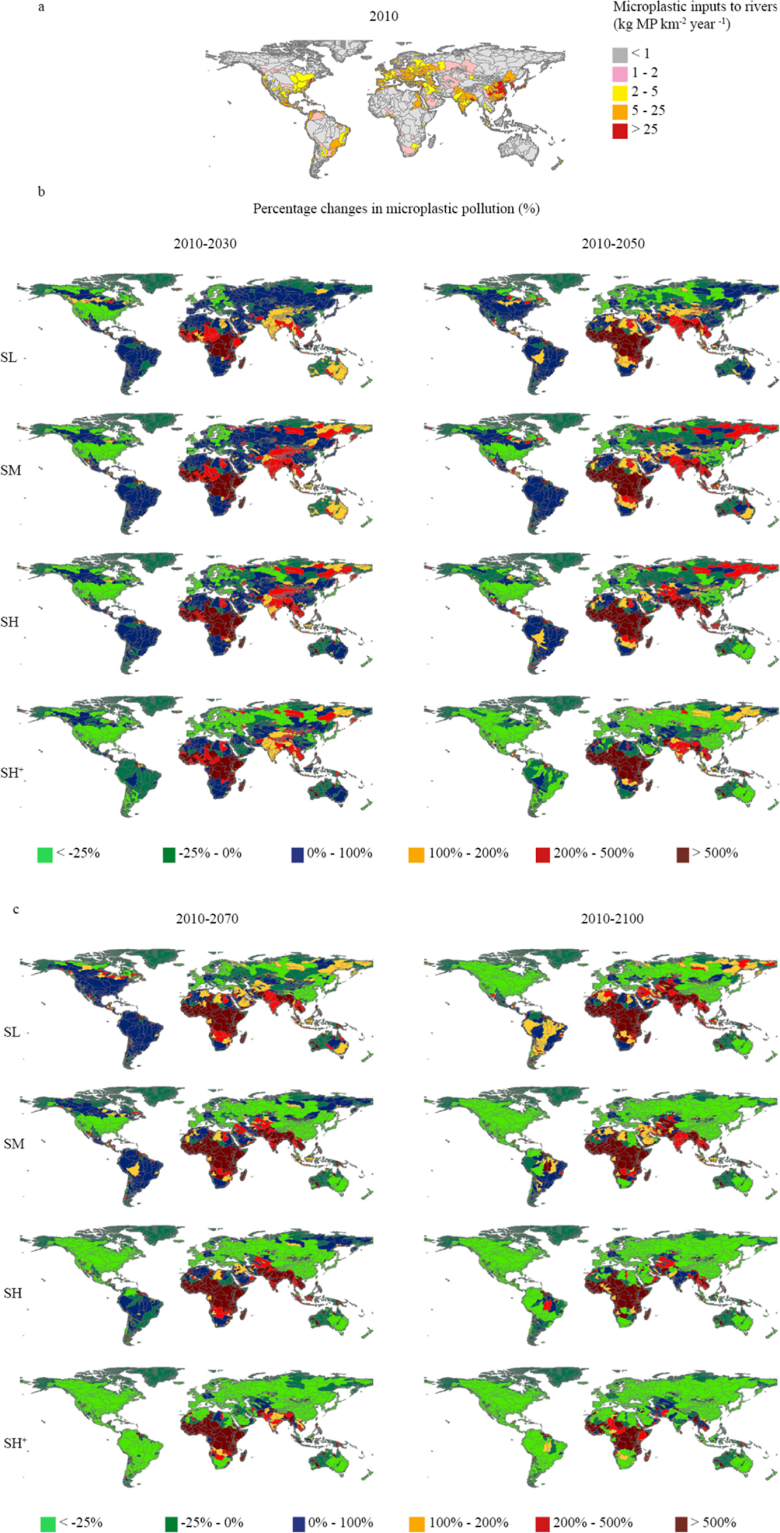
(a) Microplastic pollution in rivers (kton microplastic km^–2^ year^–1^) in 2010 in rivers of 10,226 sub-basins. (b) The changes in microplastic pollution (%) in 10,226 sub-basins according to the four scenarios between 2030 and 2050 compared to 2010. (c) The changes in microplastic pollution (%) in 10,226 sub-basins according to the four scenarios between 2070 and 2100 compared to 2010. The SL, SM, SH, and SH^+^ represent the Shared Socioeconomic Pathway 2 low, moderate, high and high plus scenarios, respectively.

In 2010, Asia was the main hotspot area for global MP pollution in rivers (Figure 3a). Many sub-basins in Southeast Asia received MP inputs of more than 5 kg km^–2^ year^–1^ in 2010. This high input of MP in rivers resulted from low removal fractions during treatment and more population connected to the sewer systems in Asia, amounting to approximately 670 million people in 2010 (Figures S3 and S5). In addition, MP inputs of more than 2 kg km^–2^ year^–1^ entered some rivers in Europe and the southern regions of North America (Figure 3a). This is explained by the high population connected to the sewer systems in Europe and these regions of North America (Figure S5).

In 2030, many African and South Asian rivers are projected to receive more than double MP inputs compared to 2010 in the four scenarios (Figure 3b). This increasing trend is expected to continue until 2050, mainly because of the high population with sewer connections in these regions (Figure S5). In 2050, MP inputs in rivers in SL and SM scenarios are projected to be higher than in the SH scenario in some sub-basins, such as those in South Asia and the Far East (Figure 3b). This is because population growth connected to the sewer systems is projected to outpace improvement in sewer treatment. However, many European and American rivers are projected to show a decreasing trend in the SH^+^ scenario in 2050 since more countries are expected to reach the 30% reduction target in these regions (Figure 3b).

In 2070, African rivers are expected to become the main hotspots in MP pollution (Figure 3c). In many African rivers, neither the combined strategy nor the technology-oriented strategy is projected to reduce MP inputs in 2070 (Figure 3c). This is associated with both the low removal fractions of MPs during treatment in Africa and the high African population connected to the sewer systems, projected to grow by at least 8-fold between 2010 and 2070 across all four scenarios (Figures S4 and S5). Besides Africa, some Southeast Asian rivers are projected to have more than double MP inputs in all scenarios, because of an increase in population with sewer connections in 2070 (Figures 3c and S5).

In 2100, many African rivers are expected to receive more than 5-fold MP inputs compared to 2010 in the four scenarios (Figure 3c). In the SL, SM, and SH scenarios, only a few sub-basins in Africa are projected to reduce MPs by 2100. For the other sub-basins, MP in rivers is projected to increase due to both increasing African population with sewer connections (at least 13 times higher) and low removal fractions during wastewater treatment in 2100 (Figures S4 and S5). However, more African sub-basins in the SH^+^ scenario are expected to exist to reduce MP inputs in 2100 than in 2010 (Figure 3c). Similarly, MP inputs to South Asian rivers in the SH^+^ scenario are projected to reduce in 2100 because more African and South Asian countries are expected to reach the 30% reduction target (Figure 3c).

### 3.3 Dominant Microplastic Sources in Rivers

**Figure 4:**
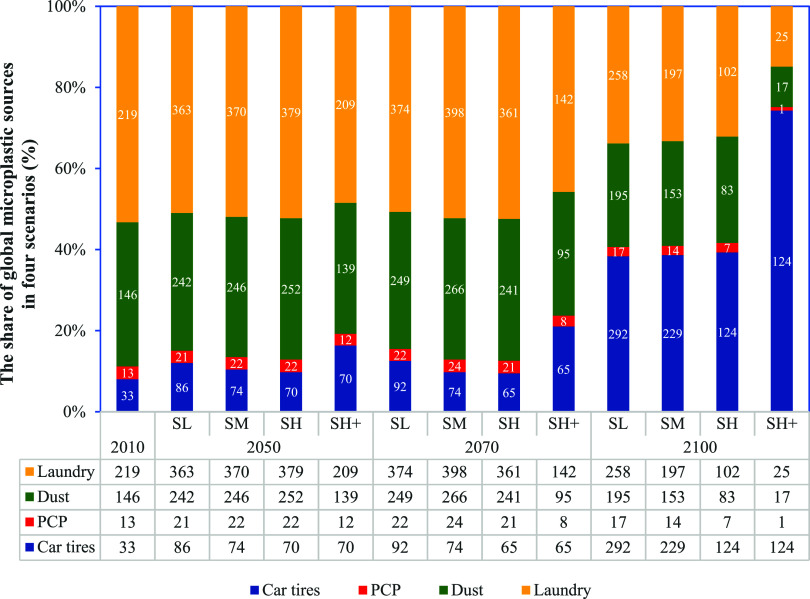
Share of four microplastic sources (%) in the SL, SM, SH, and SH^+^ scenarios in 2010, 2050, 2070, and 2100 on a global scale. The table and white numbers in the bar chart show microplastic inputs (kton per year) from different sources to global rivers in four scenarios in 2010, 2050, 2070, and 2100. The SL, SM, SH, and SH^+^ represent the Shared Socioeconomic Pathway 2 low, moderate, high and high plus scenarios, respectively. PCP represents personal care product.

**Figure 5:**
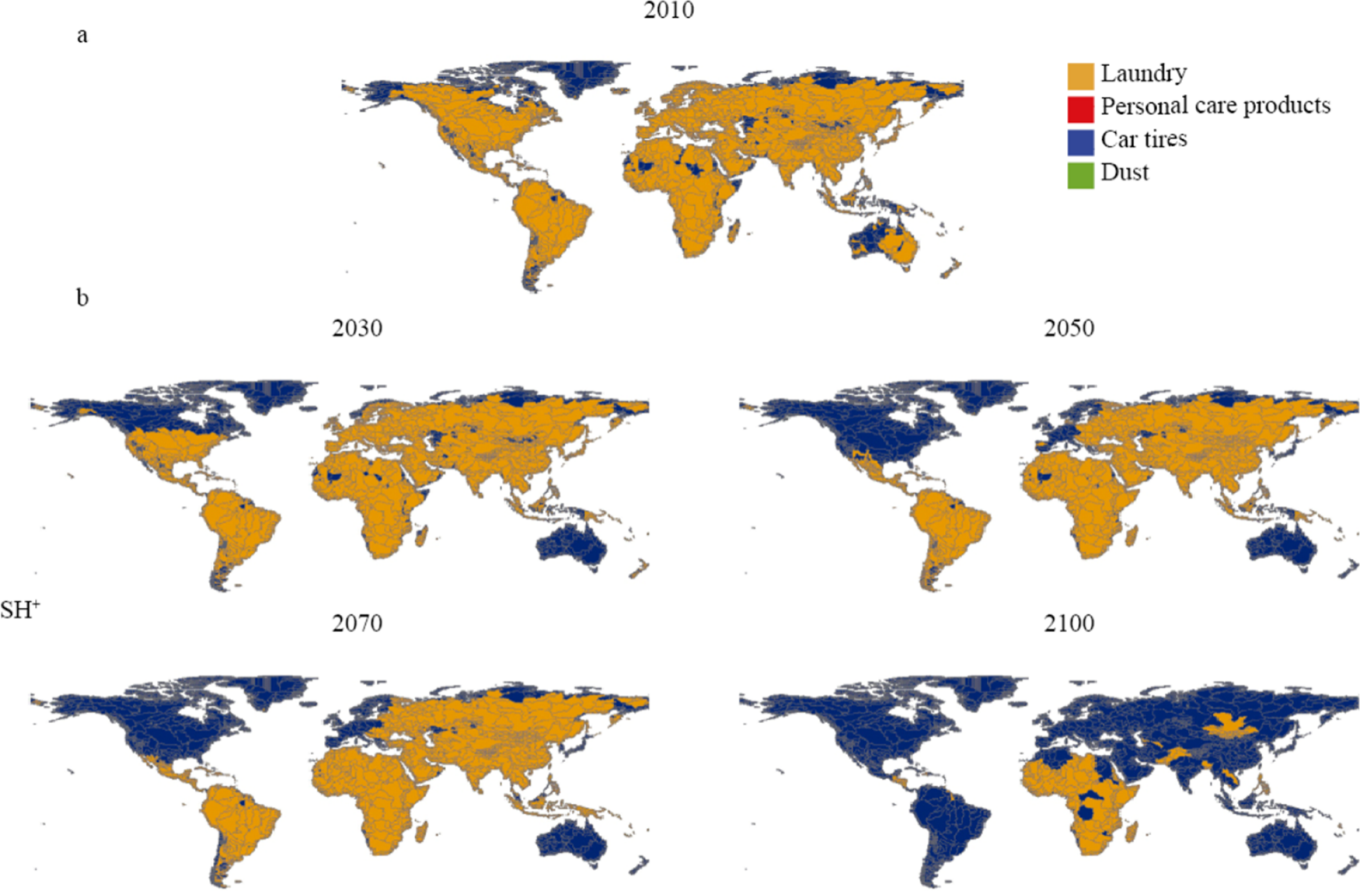
(a) Dominant microplastic sources in 10,226 sub-basins in 2010. (b) The dominant microplastic source in the SH^+^ scenario in 2030, 2050, 2070, and 2100 is presented as an illustrative example. The SH^+^ represents the Shared Socioeconomic Pathway 2 high plus scenario.

In 2010, laundry fibers were the dominant MP source worldwide, responsible for over half of the global MP pollution in rivers, amounting to 219 kton (Figure 4). This is attributed to the higher consumption rate of laundry fibers compared to other MP sources, such as PCPs and household dust (Table S4). Almost all rivers received MP primarily from laundry fibers in 2010 (Figure 5a). In addition, household dust was a significant source of MP, contributing approximately 36% on a global scale, equivalent to 146 kton, but it was not the dominant MP source in sub-basins (Figures 4 and 5a).

In 2050, laundry fibers are still projected to be a dominant source of MP in the four scenarios (Figure 4). MP inputs into rivers from all sources are expected to increase in the SL, SM and SH scenarios compared to 2010. However, in the SH^+^ scenario, only MP inputs originating from car tires are projected to increase in 2050 than in 2010. In many sub-basins in Europe and North America, the dominant MP source in rivers is projected to shift from laundry fibers to car tires by 2050 in the SH^+^ scenario (Figure 5b). This is attributed to the expected significant rise in the number of developed countries (HDI > 0.785) in these regions by 2050 compared to 2010, leading to an increase in the number of cars on the road and, consequently, greater MP production from car tires.

By 2070, dramatic differences are not expected in dominant MP sources between the four scenarios (Figure 4). Laundry fibers are projected to remain the main MP source in all scenarios by more or less 50%, similar to 2010. Nevertheless, the share of car tires in the SH^+^ scenario is projected to increase slightly in 2070 compared to 2010 globally. Australian, European and North American rivers are projected to be polluted by MP, primarily arising from car tires in 2070 in the SH^+^ scenario (Figure 5b). This is due to the higher number of developed countries in 2070, causing greater MP production from car tires.

In 2100, car tires are expected to be the dominant MP source globally (Figure 4). In all scenarios, global MP pollution in rivers originating from car tires is expected to increase significantly in 2100 compared to 2010. In the SH^+^ scenario, car tires are expected to account for approximately 75% of MP pollution, equivalent to 124 kton. Meanwhile, contributions from laundry fibers, household dust and PCPs are projected to decrease significantly than in 2010 (Figure 4). Except for some African and Asian rivers, the world’s rivers are projected to be dominated by MP inputs from car tires in 2100 in the SH^+^ scenario (Figure 5b). This is explained by the increase in the number of developed countries in 2100 than in 2010, thereby leading to a higher production of MPs from car tires as a result of the use of more cars.

## 4 Discussion

### 4.1 Model Limitations

Our model does not account for the diffuse sources resulting from the degradation of macroplastics into MPs.^3,6,36,37^ While the MARINA-Plastics model includes diffuse sources, our study specifically focuses on point sources (i.e., sewer effluents). This focus is justified, as sewer systems are responsible for more than 80% of MP pollution in rivers globally.^6^ Li et al. emphasized that sewer effluents are the major sources of MP in Chinese rivers, whereas diffuse sources such as agricultural plastic mulching are mainly responsible for macroplastics.^6,14^ Macroplastics from mismanaged solid waste may become the secondary source of MPs in some rivers of Africa and Asia as shown in Strokal et al.^6^ For those rivers, we may underestimate river pollution levels. Since our study is concerned solely with MPs, we concentrated on sewer systems as the main point source at a larger scale. Additionally, other MP source types, such as airborne MPs, aquaculture, fisheries, ships, and nurdles (i.e., plastic pellets), were not included.^38−40^ Thus, this limitation can lead to an underestimation of our results in some sub-basins where those activities are dominant.

Another limitation relates to factors that may affect MP pollution levels in rivers. Examples include surface runoff, atmospheric deposition and MP retentions in rivers.^6,41−43^ Our model does not account for these factors due to limited data. For example, runoff may transport MPs into surface waters, but data on surface runoff on a global scale are limited.^44^ As a result, our results may underestimate the true MP input into rivers.

### 4.2 Scenario Limitations

The SH^+^ scenario has some assumptions for target years (Figure S1). The Zero Pollution Action Plan justifies assuming a 30% reduction in MPs released into the environment by 2030 for countries with an HDI exceeding 0.62^27^ (Figure S1). However, the goals for other years (2040, 2060 and 2070) are arbitrary. Nevertheless, the impact of this assumption on our results is limited because MP inputs to rivers in the SH^+^ scenario are expected to be much less than in other scenarios in the future (Figure 2). Furthermore, we extrapolated the Zero Pollution Action Plan, which is applicable to European Union (EU) countries on a global scale. Nevertheless, non-European Union countries such as China and Turkey have targets and policies to mitigate MP pollution, similar to the Zero Pollution Action Plan.^27,45,46^ Hence, we believe this assumption is not unrealistic.

### 4.3 Comparison with Other Studies

Jones et al. noted that wastewater treatment improvements are insufficient to meet the SDGs in many world regions.^47^ Our research also concluded that wastewater treatment technology-oriented strategies may not be sufficient to reduce MP inputs in rivers of many sub-basins because sewer connections outpace the efficiency of MP removal in wastewater treatment plants (Section 3). In addition, Strokal et al. projected a substantial increase in MP pollution in African rivers in the 21st century compared to 2010, indicating a potential failure to achieve the SDGs.^18^ Similarly, a recent study stated that Sub-Saharan Africa will be the hotspot of surface water pollution in the future globally.^48^ van Wijnen et al. also underlined that the export of MPs to coastal seas dramatically increases in Africa when taking into account three MP sources (car tire, laundry and personal care products).^8^ Consistently, our study highlighted that MP pollution in African rivers is expected to increase in 2100 compared to 2010 (Section 3). However, our study includes more sources and additional future scenarios to predict MP inputs in global rivers.

Meijer et al. revealed a significant relationship between MP inputs into rivers and population density.^7^ Our results are in line with Meijer et al. because the main drivers in our model are the population connected to the sewer system and removal fractions during wastewater treatment (Section 2). The population connected to the sewer system is also associated with population density. Mai et al. stated that Asian rivers are the main sources of plastic outflows, though this study focused on both MP and macroplastics.^4^ Similarly Lebreton et al. concluded that Asia is the main contributor to global plastic emissions into the seas, which aligns with our results^16^ (Section 3). Nevertheless, the main difference in the estimation of MP inputs between our results and Lebreton et al. is that we focus on MPs, whereas Lebreton et al. focus on MPs as well as macroplastics. Siegfried et al. pointed out the remarkable contribution of car tires to MP pollution, which aligns with our findings, particularly regarding future MP pollution (Section 3).^1^ While Siegfried et al. only focused exclusively on European seas for the years 2000 and 2050, our study analyzes global rivers between 2010 and 2100, taking time steps of 10 years.

Additionally, we conducted an analysis of the minimum, average, and maximum global MP inputs for sub-basins in the four scenarios between 2010 and 2100 (Table S5). Although the minimum values did not change significantly between scenarios from 2010 to 2100, the average values were consistent with global MP inputs to rivers (Figure 2). This consistency suggests our model reflects general trends in MP pollution. The maximum values, however, showed substantial variation, highlighting the potential for extreme cases of MP pollution in different scenarios (Table S5). This analysis emphasized the importance of considering different scenarios when predicting future MP pollution levels. The variation in maximum values underscores the need for targeted strategies to mitigate extreme cases of MP pollution. By understanding the range of possible outcomes, policymakers can better prepare for and address the challenges of MP inputs in rivers.

### 4.4 Sewer Connection and Treatment Effects on MP Pollution

In addition to the population growth, our results are also driven by the following two factors: (1) the population connected to sewer systems and (2) the removal fractions of MP during wastewater treatment. These two factors are spatially explicit and differ among the studied 10,226 sub-basins. For example, when the population connected to sewer systems increases, MP inputs into rivers may also increase. Conversely, improvements in treatment efficiencies are expected to increase removal fractions of MP during treatment and thus remove a larger proportion of MPs from wastewater. This may result in lower MP inputs into rivers from sewer systems.

**Figure 6:**
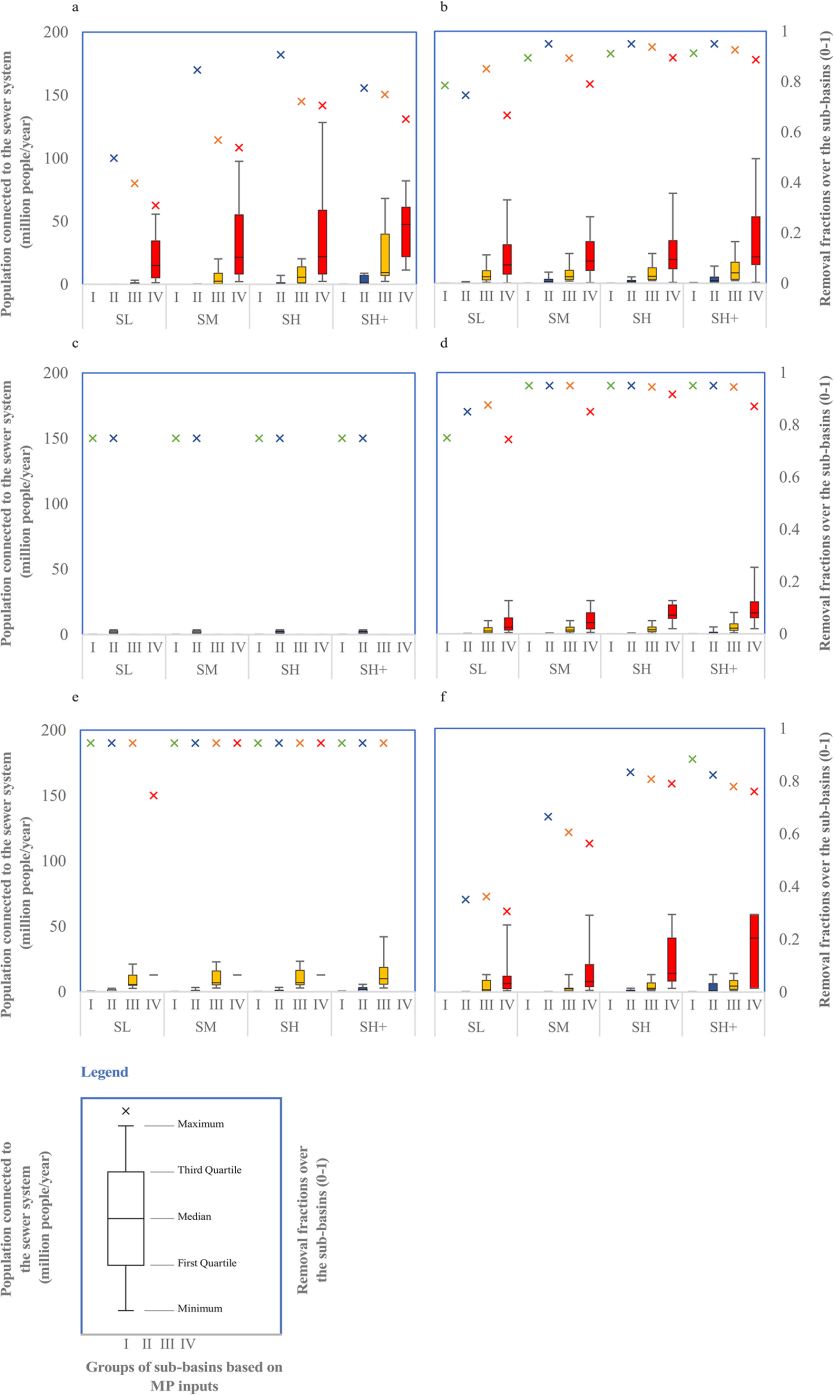
Boxplots for the population connected to sewer systems (million people per year) in (a) Africa, (b) Asia, (c) Australia, (d) Europe, (e) North America, and (f) South America over the four scenarios and among the four groups of MP inputs into rivers in 2100. The “X” marker represents the mean of removal fractions (0–1) over the sub-basins. The four groups were defined based on quantile intervals of MP inputs to rivers in all sub-basins in 2100: group I (0–25%), group II (25–50%), group III (50–75%), and group IV (75–100%).

We analyzed the interplay between the projected population with sewer connectivity and removal fractions of MPs during treatment. This analysis was conducted over the six continents under the four scenarios for the year 2100. For this, we first categorized sub-basins into four groups based on quantile intervals (25, 50, 75%) of projected MP inputs into rivers, defining them as follows: group I (0–25%), group II (25–50%), group III (50–75%), and group IV (75–100%, river pollution hotspot with high MP inputs compared to the other sub-basins). Next, we plotted the mean removal fractions over sub-basins, along with the population connected to sewer systems, segmented by these four groups (Figure 6).

The analysis indicates that MP inputs into rivers in hotspot sub-basins (group IV) are projected to increase more substantially than in the other three groups in Africa, Asia, Europe, and South America in 2100, primarily due to the expected increase in populations connected to the sewer systems within these regions. Hotspots exhibit higher median values and greater variability, suggesting that more people are expected to be connected to sewer systems in these areas. Additionally, MP removal efficiencies in these hotspots are projected to be lower compared to other groups (Figure 6). This analysis underscores the critical influence of these two driving factors in shaping MP pollution levels in rivers, consistent with our findings (see Section 3).

### 4.5 Contribution to Policy

The results may help policymakers in two ways. First, they can help determine when to focus on source-oriented or/and treatment technology-oriented strategies for MP reduction in a basin in the future. For example, African rivers are expected to receive more than 5-fold MPs in 2100 than in 2010 (Figure 3). This is a result of projected increases in population and rapid urbanization in the future. This message is a warning signal for African policy-makers to develop water pollution control strategies to avoid future pollution from increasing population and urbanization. However, there are challenges associated with the practical implementation of these strategies. Source-oriented strategies are related to environmental behaviors, education, awareness, habits etc.^49,50^ While these changes may require significant effort, their effects can be observed relatively quickly. For instance, MP emission to European rivers is expected to be much lower in the SH^+^ in 2030 than in 2010 (Figure 3). Therefore, European policymakers may prioritize source-oriented strategies to significantly reduce MPs in 2030. Conversely, the effects of treatment technology-oriented strategies cannot be seen in the near future since population growth with sewer connections is expected to outpace improvements in wastewater treatment efficiencies (Figure 3). For instance, no significant difference in MP inputs into rivers is expected between the SL and SH scenarios by 2030. However, MP inputs by 2100 are projected to be much lower in the SH scenario than in the SL scenario (Figures 2a and 3b,3c), indicating that treatment technology-oriented strategies are more effective as long-term solutions for reducing MP inputs into rivers globally. As a result, insights from our study can help policymakers prioritize reduction strategies for combating MPs in rivers.

Second, our study provides insight into which MP sources may lead to more pollution in the future. By presenting future MP pollution based on different sources, we aim to facilitate the identification of dominant sources in sub-basins (Figure 5a). Since resources are limited in the real world, it is crucial to understand which MP source is most abundant in a basin. For instance, European policymakers may prioritize reducing MP originating from car tires by 2050, since MP inputs from car tires are expected to be the dominant MP source in Europe by then (Figure 5b). In addition, wear from car tires is expected to be the dominant source of MPs globally in the future. This could encourage science to make innovations for nonplastic materials to minimize the impact of car tire wear on MP inputs in rivers.

Our results contribute to achieving SDG6 by demonstrating that reducing MP pollution in rivers directly supports clean water and sanitation efforts. Our scenarios are useful for exploring the impacts of combined wastewater treatment levels and the population with sewer connections on future MP emissions into rivers. For instance, increased sewer connectivity in combination with improved treatment, as shown in our scenarios, directly contributes to SDG6.1. Additionally, the SH and SH^+^ scenarios illustrate how the high treatment technologies lead to considerable reductions in MP pollution, thereby improving ambient water quality in alignment with SDG6.3. These findings underscore the value of implementing combined wastewater treatment and sewer connections to effectively combat MPs in rivers both in the short and long-term, aligned with SDG6.

To conclude, the results show that the combined strategy (high wastewater treatment and source-oriented measures) is expected to be the most effective for reducing future MPs in rivers on a global scale. By 2100, this combined strategy is projected to result in a 68% MP reduction compared to 2010 globally. African rivers will be the main hotspots, receiving more than five times more MPs in 2100 than in 2010. In 2100, car tire wear is expected to be the dominant source of MPs globally. Our insights can support the implementation of the European Green Deal and the realization of Sustainable Development Goal 6 (clean water).

## Supporting Information Available

Names of countries in the MARINA-Plastics model (Table S1); model equations (Table S2); model description (Table S3); derivation of model variables (Table S4); statistical analysis of variance (Table S5); changes in consumption rates in the SH^+^ scenario (Figure S1); average global removal efficiencies (Figure S2); global total population connected to sewer systems (Figure S3); average removal efficiencies by region (Figure S4); total population connected to sewer systems by region (Figure S5); Human Development Index (Figure S6), and country borders in the model (Figure S7) (PDF)

## References

[ref1] Siegfried M.; Koelmans A. A.; Besseling E.; Kroeze C. Export of Microplastics from Land to Sea. A Modelling Approach. Water Res. 2017, 127, 249–257. 10.1016/j.watres.2017.10.011. 29059612

[ref2] Wagner M.; Scherer C.; Alvarez-Muñoz D.; Brennholt N.; Bourrain X.; Buchinger S.; Fries E.; Grosbois C.; Klasmeier J.; Marti T.; Rodriguez-Mozaz S.; Urbatzka R.; Vethaak A. D.; Winther-Nielsen M.; Reifferscheid G. Microplastics in Freshwater Ecosystems: What We Know and What We Need to Know. Environ. Sci. Eur. 2014, 26 (1), 12 10.1186/s12302-014-0012-7. 28936382 PMC5566174

[ref3] Lebreton L.; Andrady A. Future Scenarios of Global Plastic Waste Generation and Disposal. Palgrave Commun. 2019, 5 (1), 6 10.1057/s41599-018-0212-7.

[ref4] Mai L.; Sun X.-F.; Xia L.-L.; Bao L.-J.; Liu L.-Y.; Zeng E. Y. Global Riverine Plastic Outflows. Environ. Sci. Technol. 2020, 54 (16), 10049–10056. 10.1021/acs.est.0c02273. 32700904

[ref5] Avio C. G.; Gorbi S.; Regoli F. Plastics and Microplastics in the Oceans: From Emerging Pollutants to Emerged Threat. Mar. Environ. Res. 2017, 128, 2–11. 10.1016/j.marenvres.2016.05.012. 27233985

[ref6] Strokal M.; Vriend P.; Bak M. P.; Kroeze C.; van Wijnen J.; van Emmerik T. River Export of Macro- and Microplastics to Seas by Sources Worldwide. Nat. Commun. 2023, 14 (1), 4842 10.1038/s41467-023-40501-9. 37563145 PMC10415377

[ref7] Meijer L. J. J.; van Emmerik T.; van der Ent R.; Schmidt C.; Lebreton L. More than 1000 Rivers Account for 80% of Global Riverine Plastic Emissions into the Ocean. Sci. Adv. 2021, 7 (18), eaaz5803 10.1126/sciadv.aaz5803. 33931460 PMC8087412

[ref8] van Wijnen J.; Ragas A. M. J.; Kroeze C. Modelling Global River Export of Microplastics to the Marine Environment: Sources and Future Trends. Sci. Total Environ. 2019, 673, 392–401. 10.1016/j.scitotenv.2019.04.078. 30991329

[ref9] Van Emmerik T.; Mellink Y.; Hauk R.; Waldschläger K.; Schreyers L. Rivers as Plastic Reservoirs. Front. Water 2022, 3, 786936 10.3389/frwa.2021.786936.

[ref10] Kataoka T.; Nihei Y.; Kudou K.; Hinata H. Assessment of the Sources and Inflow Processes of Microplastics in the River Environments of Japan. Environ. Pollut. 2019, 244, 958–965. 10.1016/j.envpol.2018.10.111. 30469290

[ref11] Eerkes-Medrano D.; Thompson R. C.; Aldridge D. C. Microplastics in Freshwater Systems: A Review of the Emerging Threats, Identification of Knowledge Gaps and Prioritisation of Research Needs. Water Res. 2015, 75, 63–82. 10.1016/j.watres.2015.02.012. 25746963

[ref12] Murphy F.; Ewins C.; Carbonnier F.; Quinn B. Wastewater Treatment Works (WwTW) as a Source of Microplastics in the Aquatic Environment. Environ. Sci. Technol. 2016, 50 (11), 5800–5808. 10.1021/acs.est.5b05416. 27191224

[ref13] Blair R. M.; Waldron S.; Phoenix V.; Gauchotte-Lindsay C. Micro- and Nanoplastic Pollution of Freshwater and Wastewater Treatment Systems. Springer Sci. Rev. 2017, 5 (1–2), 19–30. 10.1007/s40362-017-0044-7.

[ref14] Li Y.; Zhang Q.; Baartman J.; van Wijnen J.; Beriot N.; Kroeze C.; Wang M.; Xu W.; Ma L.; Wang K.; Zhang F.; Strokal M. The Plastic Age: River Pollution in China from Crop Production and Urbanization. Environ. Sci. Technol. 2023, 57, 12019–12032. 10.1021/acs.est.3c03374. 37527154 PMC10433511

[ref15] Schmidt C.; Krauth T.; Wagner S. Export of Plastic Debris by Rivers into the Sea. Environ. Sci. Technol. 2017, 51 (21), 12246–12253. 10.1021/acs.est.7b02368. 29019247

[ref16] Lebreton L. C. M.; Van Der Zwet J.; Damsteeg J.-W.; Slat B.; Andrady A.; Reisser J. River Plastic Emissions to the World’s Oceans. Nat. Commun. 2017, 8 (1), 15611 10.1038/ncomms15611. 28589961 PMC5467230

[ref17] Koelmans A. A.; Nor N. H. M.; Hermsen E.; Kooi M.; Mintenig S. M.; De France J. Microplastics in Freshwaters and Drinking Water: Critical Review and Assessment of Data Quality. Water Res. 2019, 155, 410–422. 10.1016/j.watres.2019.02.054. 30861380 PMC6449537

[ref18] Strokal M.; Bai Z.; Franssen W.; Hofstra N.; Koelmans A. A.; Ludwig F.; Ma L.; van Puijenbroek P.; Spanier J. E.; Vermeulen L. C.; van Vliet M. T. H.; van Wijnen J.; Kroeze C. Urbanization: An Increasing Source of Multiple Pollutants to Rivers in the 21st Century. npj Urban Sustainability 2021, 1 (1), 24 10.1038/s42949-021-00026-w.

[ref19] Strokal V.; Kuiper E. J.; Bak M. P.; Vriend P.; Wang M.; van Wijnen J.; Strokal M. Future Microplastics in the Black Sea: River Exports and Reduction Options for Zero Pollution. Mar. Pollut. Bull. 2022, 178, 113633 10.1016/j.marpolbul.2022.113633. 35398693

[ref20] Fältström E.; Anderberg S. Towards Control Strategies for Microplastics in Urban Water. Environ. Sci. Pollut. Res. 2020, 27 (32), 40421–40433. 10.1007/s11356-020-10064-z. PMC7546980 32666462

[ref21] Lee S.-Y.; Rhee S.-K. From End-of-Pipe Technology towards Pollution Preventive Approach: The Evolution of Corporate Environmentalism in Korea. J. Cleaner Prod. 2005, 13 (4), 387–395. 10.1016/j.jclepro.2003.10.010.

[ref22] Meng F.; Fu G.; Butler D. Water Quality Permitting: From End-of-Pipe to Operational Strategies. Water Res. 2016, 101, 114–126. 10.1016/j.watres.2016.05.078. 27262116

[ref23] Zotter K. A. End-of-Pipe” versus “Process-Integrated” Water Conservation Solutions. J. Cleaner Prod. 2004, 12 (7), 685–695. 10.1016/S0959-6526(03)00115-X.

[ref24] Westphalen H.; Abdelrasoul A. Challenges and Treatment of Microplastics in Water. In Water Challenges of an Urbanizing World; Glavan M., Ed.; InTech, 2018.

[ref25] Belayutham S.; González V. A.; Yiu T. W. A Cleaner Production-Pollution Prevention Based Framework for Construction Site Induced Water Pollution. J. Cleaner Prod. 2016, 135, 1363–1378. 10.1016/j.jclepro.2016.07.003.

[ref26] Dvorak B. Pollution Prevention/Environmental Sustainability for Industry. Environments 2021, 8 (9), 91 10.3390/environments8090091.

[ref27] EU Action Plan: “Towards Zero Pollution for Air, Water and Soil; European Commission, 2021.

[ref28] van Puijenbroek P.; Beusen A. H. W.; Bouwman A. F.; Ayeri T.; Strokal M.; Hofstra N. Quantifying Future Sanitation Scenarios and Progress towards SDG Targets in the Shared Socioeconomic Pathways. J. Environ. Manage. 2023, 346, 118921 10.1016/j.jenvman.2023.118921. 37738725

[ref29] Jones B.; O’Neill B. C. Spatially Explicit Global Population Scenarios Consistent with the Shared Socioeconomic Pathways. Environ. Res. Lett. 2016, 11 (8), 084003 10.1088/1748-9326/11/8/084003.

[ref30] Strokal M.; Spanier J. E.; Kroeze C.; Koelmans A. A.; Flörke M.; Franssen W.; Hofstra N.; Langan S.; Tang T.; van Vliet M. T.; Wada Y.; Wang M.; van Wijnen J.; Williams R. Global Multi-Pollutant Modelling of Water Quality: Scientific Challenges and Future Directions. Curr. Opin. Environ. Sustainability 2019, 36, 116–125. 10.1016/j.cosust.2018.11.004.

[ref31] Guo Y.; Ayeri T.; van Puijenbroek P.; Strokal M. Microplastics in Global Rivers: Sustainable Practices Sustainable Dev. 2024 10.1002/sd.3279.

[ref32] O’Neill B. C.; Kriegler E.; Ebi K. L.; Kemp-Benedict E.; Riahi K.; Rothman D. S.; van Ruijven B. J.; van Vuuren D. P.; Birkmann J.; Kok K.; Levy M.; Solecki W. The Roads Ahead: Narratives for Shared Socioeconomic Pathways Describing World Futures in the 21st Century. Global Environ. Change 2017, 42, 169–180. 10.1016/j.gloenvcha.2015.01.004.

[ref33] Van Puijenbroek P. J. T. M.; Beusen A. H. W.; Bouwman A. F. Global Nitrogen and Phosphorus in Urban Waste Water Based on the Shared Socio-Economic Pathways. J. Environ. Manage. 2019, 231, 446–456. 10.1016/j.jenvman.2018.10.048. 30368155

[ref34] Cuaresma J. C.; Lutz W. The demography of human development and climate change vulnerability: A projection exercise. Vienna Yearb. Popul. Res. 2016, 1, 241–262. 10.1553/populationyearbook2015s241.

[ref35] United Nations Development Programme Human Development Report; United Nations, 2020.

[ref36] Waldschläger K.; Lechthaler S.; Stauch G.; Schüttrumpf H. The Way of Microplastic through the Environment—Application of the Source-Pathway-Receptor Model (Review). Sci. Total Environ. 2020, 713, 136584 10.1016/j.scitotenv.2020.136584. 32019016

[ref37] Weinstein J. E.; Crocker B. K.; Gray A. D. From Macroplastic to Microplastic: Degradation of High-density Polyethylene, Polypropylene, and Polystyrene in a Salt Marsh Habitat. Environ. Toxicol. Chem. 2016, 35 (7), 1632–1640. 10.1002/etc.3432. 26992845

[ref38] Dris R.; Gasperi J.; Saad M.; Mirande C.; Tassin B. Synthetic Fibers in Atmospheric Fallout: A Source of Microplastics in the Environment?. Mar. Pollut. Bull. 2016, 104 (1–2), 290–293. 10.1016/j.marpolbul.2016.01.006. 26787549

[ref39] Tian Y.; Yang Z.; Yu X.; Jia Z.; Rosso M.; Dedman S.; Zhu J.; Xia Y.; Zhang G.; Yang J.; Wang J. Can We Quantify the Aquatic Environmental Plastic Load from Aquaculture?. Water Res. 2022, 219, 118551 10.1016/j.watres.2022.118551. 35561617

[ref40] Cai L.; Wang J.; Peng J.; Tan Z.; Zhan Z.; Tan X.; Chen Q. Characteristic of Microplastics in the Atmospheric Fallout from Dongguan City, China: Preliminary Research and First Evidence. Environ. Sci. Pollut. Res. 2017, 24 (32), 24928–24935. 10.1007/s11356-017-0116-x. 28918553

[ref41] Fahrenfeld N. L.; Arbuckle-Keil G.; Beni N. N.; Bartelt-Hunt S. L. Source Tracking Microplastics in the Freshwater Environment. TrAC, Trends Anal. Chem. 2019, 112, 248–254. 10.1016/j.trac.2018.11.030.

[ref42] Li C.; Busquets R.; Campos L. C. Assessment of Microplastics in Freshwater Systems: A Review. Sci. Total Environ. 2020, 707, 135578 10.1016/j.scitotenv.2019.135578. 31784176

[ref43] Wang C.; O’Connor D.; Wang L.; Wu W.-M.; Luo J.; Hou D. Microplastics in Urban Runoff: Global Occurrence and Fate. Water Res. 2022, 225, 119129 10.1016/j.watres.2022.119129. 36170770

[ref44] Akdogan Z.; Guven B. Microplastics in the Environment: A Critical Review of Current Understanding and Identification of Future Research Needs. Environ. Pollut. 2019, 254, 113011 10.1016/j.envpol.2019.113011. 31404735

[ref45] Çevik C.; Kıdeyş A. E.; Tavşanoğlu Ü. N.; Kankılıç G. B.; Gündoğdu S. A Review of Plastic Pollution in Aquatic Ecosystems of Turkey. Environ. Sci. Pollut. Res. 2022, 29 (18), 26230–26249. 10.1007/s11356-021-17648-3. 34853999

[ref46] Sun Y.; Liu S.; Wang P.; Jian X.; Liao X.; Chen W. Q. China’s Roadmap to Plastic Waste Management and Associated Economic Costs. J. Environ. Manage. 2022, 309, 114686 10.1016/j.jenvman.2022.114686. 35189513

[ref47] Jones E. R.; Bierkens M. F. P.; Wanders N.; Sutanudjaja E. H.; van Beek L. P. H.; van Vliet M. T. H. Current Wastewater Treatment Targets Are Insufficient to Protect Surface Water Quality. Commun. Earth Environ. 2022, 3 (1), 221 10.1038/s43247-022-00554-y.

[ref48] Jones E. R.; Bierkens M. F. P.; van Puijenbroek P. J. T. M.; van Beek L. P. H.; Wanders N.; Sutanudjaja E. H.; van Vliet M. T. H. Sub-Saharan Africa Will Increasingly Become the Dominant Hotspot of Surface Water Pollution. Nat. Water 2023, 1 (7), 602–613. 10.1038/s44221-023-00105-5.

[ref49] Heidbreder L. M.; Bablok I.; Drews S.; Menzel C. Tackling the Plastic Problem: A Review on Perceptions, Behaviors, and Interventions. Sci. Total Environ. 2019, 668, 1077–1093. 10.1016/j.scitotenv.2019.02.437. 31018449

[ref50] Soares J.; Miguel I.; Venâncio C.; Lopes I.; Oliveira M. Public Views on Plastic Pollution: Knowledge, Perceived Impacts, and pro-Environmental Behaviours. J. Hazard. Mater. 2021, 412, 125227 10.1016/j.jhazmat.2021.125227. 33951864

