# Supplementary material for: Combined Effects of Treatment and Sewer Connections to Reduce Future Microplastic Emissions in Rivers
Source: Environ Sci Technol. 2024 Nov 25;58(49):21770–81. doi: 10.1021/acs.est.4c07730 (PMC11636226; doi:10.1021/acs.est.4c07730)
Supplement: Supplementary file 1 — es4c07730_si_001.pdf [file es4c07730_si_001.pdf]

Supporting Information for:

Combined Effects of Treatment and Sewer Connections to Reduce Future Microplastic Emissions in Rivers

Tolga Ayeri[1], Yutong Guo[2], Peter J. T. M. van Puijenbroek[3], Nynke Hofstra[4], Ad M. J. Ragas[1], and Maryna Strokal[4*]*

1: Department of Environmental Science, Radboud Institute for Biological and Environmental Sciences (RIBES), Radboud University Nijmegen, 6500GL, Nijmegen, The Netherlands

2: Aquatic Ecology and Water Quality Management Group, Wageningen University and Research, Droevendaalsesteeg 4, 6708 PB, Wageningen, The Netherlands

3: PBL Netherlands Environmental Assessment Agency, PO Box 30314, 2500, GH The Hague, The Netherlands

4: Earth Systems and Global Change Group, Wageningen University and Research, Droevendaalsesteeg 4, 6708 PB, Wageningen, The Netherlands

*corresponding authors: tolga.ayeri@ru.nl and maryna.strokal@wur.nl

Supporting information summary:

Number of Pages: 17

Number of Figures: 7

Number of Tables: 5

Table of Contents

Table S1. The name of the countries used in the MARINA-Plastics model. ... S3

Table S2. The MARINA-Plastics model equations to quantify the inputs of microplastic to rivers from the sewer system in 10,226 sub-basins. ... S5

Table S3. Description of the MARINA-Plastics model variables in Table S1 ... S6

Table S4. Description of how model variables were derived for Tables S2 and S3. ... S7

Figure S1. The changes in the consumption rates of MP depending on HDI in the SH^+ scenario ... S8

Figure S2. Average global removal efficiencies of microplastic during the wastewater treatment for ten-year periods of between 2010 and 2100 according to three scenarios. ... S10

Figure S3. The total population connected to the sewer system for ten-year periods of between 2010 and 2100 according to three scenarios. ... S11

Figure S4. Average removal efficiency of microplastic in (a) African, (b) Asian, (c) Australian, (d) European, (e) North American and (f) South American for the period of 10 years between 2010 and 2100 according to three sanitation scenarios ... S11

Figure S5. (a) African, (b) Asian, (c) Australian, (d) European, (e) South American and (f) North American population connected to the sewer system for the period of 10 years between 2010 and 2100 according to three sanitation scenarios. ... S13

Figure S6. Human Development Index (HDI) for the SM in 2010, 2070 and 2100 at sub-basins. ... S14

Figure S7. The borders of countries used in this research. ... S15

Table S5. The minimum, average and maximum MP inputs in global rivers in four scenarios between 2010 and 2100 ... S16

REFERENCES ... S17

Table S1. The name of the countries used in the MARINA-Plastics model[1].

Afghanistan	Egypt	Latvia	Samoa
Albania	El Salvador	Lebanon	Saudi Arabia
Algeria	Equatorial Guinea	Liberia	Senegal
Angola	Eritrea	Libyan Arab Jamahiriya	Serbia
Argentina	Estonia	Lithuania	Sierra Leone
Armenia	Ethiopia	Luxembourg	Slovakia
Australia	Falklands Islands (Malvinas)	Macedonia. F. Yug. Rep. of	Slovenia
Austria	Fiji	Madagascar	Solomon Islands
Azerbaijan	Finland	Malawi	Somalia
Bahamas	France	Malaysia	South Africa
Bangladesh	French Guiana	Mali	South Sudan
Belarus	Gabon	Mauritania	Spain
Belgium	Gambia	Mauritius	Sri Lanka
Belize	Georgia	Mexico	Sudan
Benin	Germany	Moldova	Suriname
Bhutan	Ghana	Mongolia	Svalbard and Jan Mayen
Bolivia	Greece	Montenegro	Swaziland
Bosnia and Herzegovina	Greenland	Morocco	Sweden
Botswana	Guadeloupe	Mozambique	Switzerland
Brazil	Guatemala	Myanmar	Syria
Brunei Darussalam	Guinea	Namibia	Taiwan. Province of China
Bulgaria	Guinea-Bissau	Nepal	Tajikistan
Burkina Faso	Guyana	Netherlands	Tanzania
Burundi	Haiti	New Caledonia	Thailand
Cambodia	Honduras	New Zealand	Togo
Cameroon	Hong Kong	Nicaragua	Trinidad and Tobago
Canada	Hungary	Niger	Tunisia
Caspian Sea	Iceland	Nigeria	Turkey

Central African Republic	India	Northern Cyprus	Turkmenistan
Chad	Indonesia	Norway	Turks and Caicos Islands
Chile	Iran	Oman	Uganda
China	Iraq	Pakistan	Ukraine
Colombia	Ireland	Palestina	United Arab Emirates
Comoros	Israel	Panama	United Kingdom
Congo	Italy	Papua New Guinea	United States
Congo. The Dem. Rep. of the	Jamaica	Paraguay	Uruguay
Costa Rica	Japan	Peru	Uzbekistan
Cote d'Ivoire	Jersey	Philippines	Vanuatu
Croatia	Jordan	Poland	Venezuela
Cuba	Kazakstan	Portugal	Viet Nam
Cyprus	Kenya	Puerto Rico	Virgin Islands. U.S.
Czech Republic	Korea. Dem. People's Rep. of	Qatar	Western Sahara
Denmark	Korea. Republic of	Reunion	Yemen
Djibouti	Kosovo	Romania	Zambia
Dominican Republic	Kuwait	Russian Federation	Zimbabwe
East Timor	Kyrgyzstan	Rwanda	Åland Island
Ecuador	Lao People's Dem. Rep.	Saint Vincent & the Grenadines	

Table S2. The MARINA-Plastics model equations to quantify the inputs of microplastic to rivers from the sewer system in 10,226 sub-basins. Variables and abbreviations are explained in Table S3. Sources are given in Table S4. Source: an updated version of MARINA-Plastics based on Strokal et al., (2021)

Equations	Equations (Eq.)
$RS_{sew.j} = Pop_{sew.j} \cdot WShw_{cap.j} \cdot (1 - hw_{frem.j})$	Eq.1
$Pop_{sew.j} = Pop_{sew.urb.j} + Pop_{sew.rur.j}$	Eq.2
$Pop_{sew.urb.j} = Pop_{urb.j} \cdot fr_{pop.sew.urb.j}$	Eq.3
$Pop_{sew.rur.j} = Pop_{rur.j} \cdot fr_{pop.sew.rur.j}$	Eq.4
$WShw_{cap.j} = WShw_{cap.tyres.j} + WShw_{cap.pcp.j} + + WShw_{cap.ldry.j} + WShw_{cap.dust.j}$	Eq.5
$hw_{frem.P.j} = (f_{p.j} \cdot RE_p) + (f_{s.j} \cdot RE_s) + (f_{t.j} \cdot RE_t) + (f_{q.j} \cdot RE_q) + (f_{no.j} \cdot RE_{no})$ *for only phosphorus (P) (See Table S4)*	Eq.6*
$hw_{frem.j}$ are based on $hw_{frem.P.j}$ (See Table S4)	-

*We calculate the removal efficiencies for phosphorus by country, then downscale to grids and sub-basins (see code f in Table S4). It includes four types of the treatment: primary, secondary, tertiary and quaternary based on van Puijenbroek et al. (2023)[2]. The quaternary treatment was not considered in the original MARINA-Plastics model[1] (Table S5).

Table S3. Description of the MARINA-Plastics model variables in Table S1[1]. Sources are given in Table S4.

Variable	Description	Unit	Equation in Table S2	Processing category (Table S4)
$RS_{sew.j}$	Microplastic input to rivers in sub-basin j from sewer systems	kg year^{-1}	Eq. 1	-
$Pop_{sew.j}$	Population in sub-basin j with sewer connection.	People year^{-1}	Eq. 2	-
$WShw_{cap.j}$	The consumption rate of microplastics per capita in sub-basin j	kg capita^{-1} year^{-1}	Eq.1	-
$hw_{frem.j}$	The removal fraction of microplastics during the wastewater treatment in sub-basin j	0-1	Eq. 1 - 6	-
$Pop_{sew.urb.j}$, $Pop_{sew.rur.j}$	Urban and rural population in sub-basin j with sewer connections, respectively	People year^{-1}	Eq. 2-3-4	-
$Pop_{urb.j}$, $Pop_{rur.j}$	Urban and rural population in sub-basin j, respectively	People year^{-1}	Eq. 3-4	a
$fr_{pop.sew.urb.j}$, $fr_{pop.sew.rur.j}$	Fractions of urban and rural population in sub-basin j with sewer connections, respectively	0-1	Eq. 3-4	b
$WShw_{cap.tyres.j}$, $WShw_{cap.pcp.j}$, $WShw_{cap.ldry.j}$, $WShw_{cap.dust.j}$	The consumption rates per capita of microplastic resulting from car tyres (tyres), personal care products (pcp), laundry (ldry) and household dust (dust), respectively	kg capita^{-1} year^{-1}	Eq. 5	c
$RE_{p.j}$,$RE_{s.j}$,$RE_{t.j}$,$RE_{q.j}$, $RE_{no.j}$	Removal efficiencies of microplastic in sub-basin j during primary, secondary, tertiary, quaternary treatment and without treatment, respectively	0-1	Eq. 6	e
$f_{p.j}$, $f_{s.j}$, $f_{t.j}$,$f_{q.j}$, $f_{no.j}$	Distribution of treatment types in sub-basin j among primary, secondary, tertiary, quaternary and no treatment, respectively	0-1	Eq. 6	f

Table S4. Description of how model variables were derived for Tables S2 and S3. SSP2 represents the Shared Socio-economic Pathway 2. HDI represents Human Development Index[3]. Source: The updated version of MARINA-Plastics based on Strokal et al., (2021)

Code in Table S3	Explanation and sources of data
a	Urban and rural population for 2010, 2020, 2030, 2040, 2050, 2060, 2070, 2080, 2090 and 2100 was directly taken from the NCAR dataset of Jones & O'Neill (2016). The data were available at 0.125 degree cell. We aggregated the data to 0.5° grid and then to sub-basins. We summed gridded values over the corresponding sub-basins in ArcGIS.
b	The fraction of urban and rural population with sewer system were available by country for ten-year time steps of 2010-2100 from van Puijenbroek et al. (2023) and based largely on the information from the Joint Monitoring Program (https://washdata.org/) on sanitation types and other literature. We completed the dataset of van Puijenbroek et al. (2023) by adding the fractions for small countries with missing data[2]. Countries with missing data cover <10% of the total study area. We added the data for those countries using the averaged fractions for the regions covering those countries. The averaged fractions for the regions were estimated using the fractions of the countries for which data were available from van Puijenbroek et al. (2023)[2].<br><br>In order to process data from country to sub-basin scale, four steps are performed for the urban population:<br><br>Country values (0-1) are assigned to grids of 0.5˚. Every grid has the value of a country to which this grid belongs to;<br><br>Gridded values (0-1) are multiplied by the population per grid of 0.5˚ (people/year) to get the number of urban people connected to sewer per grid (people/year);<br><br>Total values (people/year) per grid are summed over the grids for each sub-basin to get the number of urban people connected to sewer per sub-basin (people/year);<br><br>The number of urban people connected to sewer per sub-basin (people/year) is divided by the total population per sub-basin (people/year) to get the fraction of urban people connected to sewer per sub-basin.<br><br>The same steps also did for the rural population.
c	Consumption rates of microplastics (MP) associated with the use of car tyres (tyres), personal care products (PCP), and from laundry (ldry) and household dust were derived directly from Siegfried et al. (2017) with modification for tyres. Values of Siegfried et al. (2017) for microplastics from personal care products, laundry and household dusts are largely based on literature review. For tyres, Strokal et al. (2019) distinguish between developing and developed countries. In the SH^+ scenario, the consumption rates were reduced for the years between 2010 and 2100 depending on HDI (See Section 2.2).

Table S4.a. The consumption rates per capita of microplastic resulting from personal care products in the SH^+ scenario (kg/cap/year). The consumption rates have been reduced based on HDI for the years (2010-2100) according to the EU Zero Pollution Action Plan (European Commission, 2021) (See Section 2.2).

$WShw_{cap.pcp.j}$	2010	2020	2030	2040	2050	2060	2070	2080	2090	2100
HDI > 0.62	0.0071	0.0059	0.0050	0.0041	0.0035	0.0029	0.0024	0.0020	0.0017	0.0014
0.62 - 0.55	0.0071	0.0063	0.0056	0.0050	0.0044	0.0039	0.0035	0.0031	0.0027	0.0024
0.55 - 0.42	0.0071	0.0066	0.0062	0.0057	0.0053	0.0050	0.0046	0.0043	0.0040	0.0037
HDI < 0.42	0.0071	0.0067	0.0063	0.0059	0.0056	0.0053	0.0050	0.0047	0.0044	0.0041

Table S4.b. The consumption rates per capita of microplastic resulting from household dust in the SH^+ scenario (kg/cap/year). The consumption rates have been reduced based on HDI for the years (2010-2100) according to the EU Zero Pollution Action Plan (European Commission, 2021) (See Section 2.2).

$WShw_{cap.dust.j}$	2010	2020	2030	2040	2050	2060	2070	2080	2090	2100
HDI > 0.62	0.0800	0.0669	0.0559	0.0467	0.0391	0.0327	0.0273	0.0228	0.0191	0.0160
0.62 - 0.55	0.0800	0.0710	0.0630	0.0559	0.0496	0.0440	0.0390	0.0346	0.0307	0.0273
0.55 - 0.42	0.0800	0.0745	0.0693	0.0646	0.0601	0.0560	0.0521	0.0485	0.0452	0.0420
HDI < 0.42	0.0800	0.0754	0.0710	0.0669	0.0630	0.0593	0.0559	0.0527	0.0496	0.0467

Table S4.c. The consumption rates per capita of microplastic resulting from laundry in the SH^+ scenario (kg/cap/year). The consumption rates have been reduced based on HDI for the years (2010-2100) according to the EU Zero Pollution Action Plan (European Commission, 2021) (See Section 2.2).

$WShw_{cap.ldry.j}$	2010	2020	2030	2040	2050	2060	2070	2080	2090	2100
HDI > 0.62	0.1200	0.1003	0.0839	0.0701	0.0586	0.0490	0.0410	0.0342	0.0286	0.0239
0.62 - 0.55	0.1200	0.1065	0.0945	0.0838	0.0744	0.0660	0.0586	0.0520	0.0461	0.0409
0.55 - 0.42	0.1200	0.1117	0.1040	0.0968	0.0902	0.0839	0.0781	0.0727	0.0677	0.0631
HDI < 0.42	0.1200	0.1130	0.1065	0.1003	0.0945	0.0890	0.0838	0.0790	0.0744	0.0701

Table S4.d. The consumption rates of microplastic sources in all scenarios.

Microplastic sources	SL	SM	SH	SH^+
Personal care products	0.071 kg/cap/year 7			Table S4.a
Household dust	0.08 kg/cap/ year 7			Table S4.b
Laundry	0.12 kg/cap/ year 7			Table S4.c
Car tyres	0.18 kg/cap/year when HDI>0.785 and 0.018 kg/cap/year when HDI<0.785 based on Siegfried et al. (2017) and Strokal et al. (2019)			

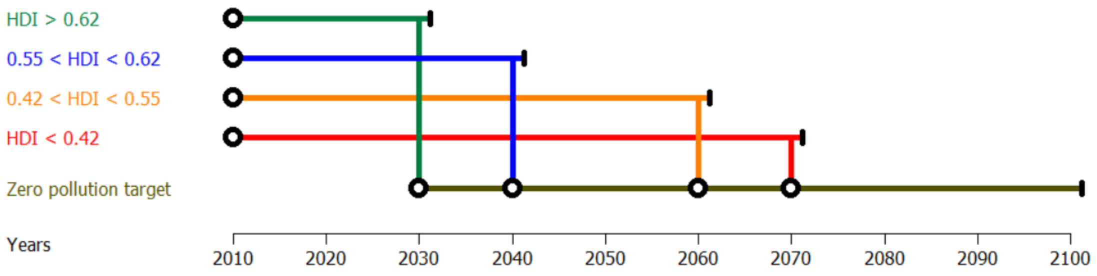

Figure S1. The changes in the consumption rates of MP depending on HDI in the SH^+ scenario. An example of how to interpret: suppose HDI in sub-basin A lower than 0.42. This sub-basin A follows the red line till 2070. This means that the sub-basin will achieve a 30% MP reduction target, specified in EU Zero Pollution Action Plan, by 2070. After 2070, it continues to follow the EU Zero Pollution Action Plan shown with dark green line until 2100[8]. HDI classifications are based on HDI fixed cut-off points which are derived from the quartiles of HDI values.

For the years between 2010 and 2100, we used these values except for car tyres where the values may change depending on HDI for all scenarios. Consumption rates are assigned to sub-basins directly.

e Removal efficiencies of pollutant i during primary, secondary, tertiary, quaternary treatment and without treatment, were directly derived from existing literature for phosphorus.

The removal efficiencies of phosphorus are (%):

primary: 10

secondary: 50.5

tertiary: 95.05

quaternary: 97.75

no treatment: 0

The removal rates of different pollutants in wastewater treatment plants depend largely on the type of pollution. In this research, primary treatment implies technologies having the removal efficiencies of microplastic <10% (see above). Examples for nutrients are screening processes to remove large particles by sedimentation and separation. Secondary treatment is with technologies having the removal efficiencies of the phosphorus 50%. This can be biologically-driven technologies. Examples are aeration by microbes to remove dissolved organic matters, and thus nutrients. Microbes can help chemicals to degrade depending how active microbes are. Tertiary treatment includes advanced technologies and chemicals to clean water further. Examples are efficient ultrafiltration methods reverse osmosis can reduce microplastics[9]. Quaternary treatment will be possible with the availability of new wastewater

	treatment technologies as an additional advanced treatment method, particularly in high-income countries[10]. Thus, quaternary treatment has a higher removal efficiency than the tertiary treatment.	
f	Distribution of treatment types among primary, secondary, tertiary, quaternary and no treatment were available by country for ten-year time steps of 2010-2100 from van Puijenbroek et al. (2023)[2]. We completed the dataset by adding the information for small countries with missing data (occupy <10% of the total study area).	

To calculate the fraction of removed phosphorus during treatment in sewer system of sub-basin j ($hw_{frem.P.j}$), six steps are performed for phosphorus:

The fraction of removed phosphorus is calculated for each country as (0-1):

(the fraction of primary treatment in each country * removal efficiencies of primary treatment) +

(the fraction of secondary treatment in each country * removal efficiencies of secondary treatment) +

(the fraction of tertiary treatment in each country * removal efficiencies of tertiary treatment) +

(the fraction of quaternary treatment in each country * removal efficiencies of quaternary treatment)+

(the fraction of no treatment in each country * removal efficiencies of no treatment);

Country values from step 1 (0-1) are assigned to grids of 0.5˚ (see code b);

Gridded values from step 2 (0-1) are multiplied by the amount of phosphorus in human waste that goes to sewer systems per grid (kg/year, see step 5.3) to get the amount of phosphorus that is treated per grid (kg/year);

Gridded values from step 3 are summed over the grids for sub-basins to get the amount of phosphorus that is treated per sub-basin (kg/year);

The amount of phosphorus in human waste that goes to sewer systems per sub-basins is calculated as:

The excretion rate per sub-basin is calculated (see code b)

Sub-basin values from step 5.1 are assigned to grids of 0.5˚ similar to the fraction of urban and rural population (see code b)

Gridded values from step 5.2 (kg/cap/year) are multiplied by the total people connected to sewer systems per grid (people/year) to get the amount of phosphorus in human waste that goes to sewer systems per grid (kg/year). Total people connected to sewer systems per grid is calculated similar to the approach for urban and rural people as described above;

Gridded values from step 5.3 (kg/year) are summed over the grids for sub-basins to get the total amount of phosphorus in human waste that goes to sewer systems

The fraction of removed phosphorus during treatment per sub-basin is calculated by dividing the amount of phosphorus that is treated per sub-basin (kg/year, step 4) by the amount of phosphorus in human waste that goes to sewer systems per sub-basin (kg/year, step 5).

Strokal et al. (2019) used the known removal rate of phosphorus to assume removal of microplastics. Siegfried et al., (2017) and van Wijnen et al., (2019) made four microplastics removal classes and related those to the known average phosphorus removal in each sub-basin. These classes represent an average microplastics removal in each sub-basin. Microplastics removal depends on size and density of the microplastics. Therefore, the removal at each individual Waste Water Treatment Plants will be dependent on these and other characteristics. In this research, on a global scale, we chose to assume average removal for each sub-basin. This results in the following removal rates of microplastics for sub-basins[7].

Table S4.e. The relationship between the removal rates for MP and phosphorus to calculate MP inputs in rivers[7] (Table S2 Eq.6)

Known phosphorus removal rates per sub-basin ($hw_{frem.P.j}$, 0-1)	Related microplastic removal rates per sub-basin ($hw_{frem.j}$, 0-1)
0-0.10	0
0.10-0.40	0.25
0.40-0.60	0.50
0.60-0.80	0.75
0.80-1.00	0.95

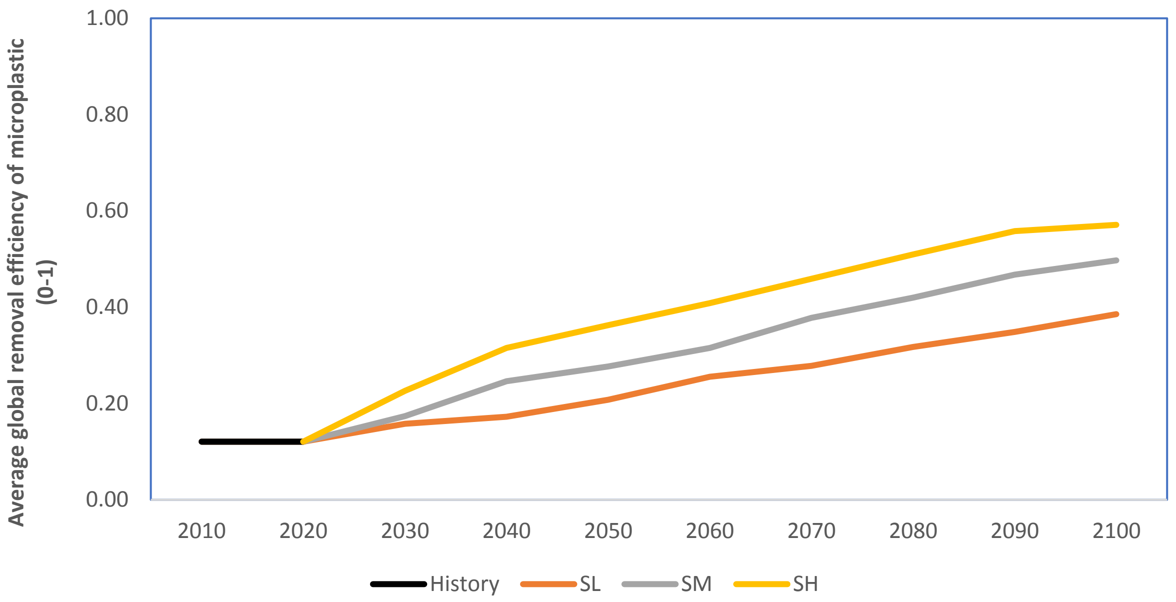

Figure S2. Average global removal efficiencies of microplastic during the wastewater treatment for ten-year periods of between 2010 and 2100 according to three scenarios. Unit is between 0 and 1. The SL, SM and SH represent the Shared Socioeconomic Pathway 2 low, moderate and high scenarios, respectively. The SH^+ scenario is not shown in the figure because it has the same removal efficiency as the SH[1]. Source: See Tables S2-S4 for the model descriptions and the scenario description; van Puijenbroek et al. (2023) , van Puijenbroek et al., (2019) and (Strokal et al., 2021).

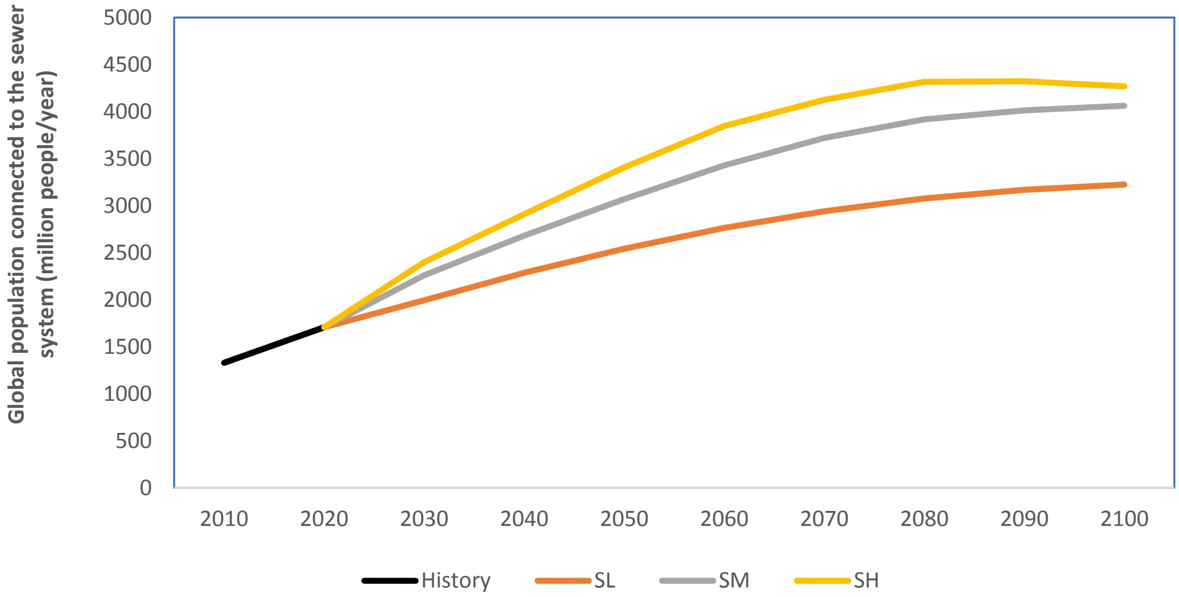

Figure S3. The total population connected to the sewer system for ten-year periods of between 2010 and 2100 according to three scenarios. As the SH^+ scenario has the same population data as the SH scenario, only the SH scenario is shown. Unit is million people per year. The SL, SM and SH represent the Shared Socioeconomic Pathway 2 low, moderate and high scenarios, respectively. The total population connected to the sewer systems in sub-basins are summed for each scenario, separately[1]. Source: See Tables S2-S4 for the model descriptions and the scenario description; van Puijenbroek et al. (2023), van Puijenbroek et al., (2019) and (Strokal et al., 2021).

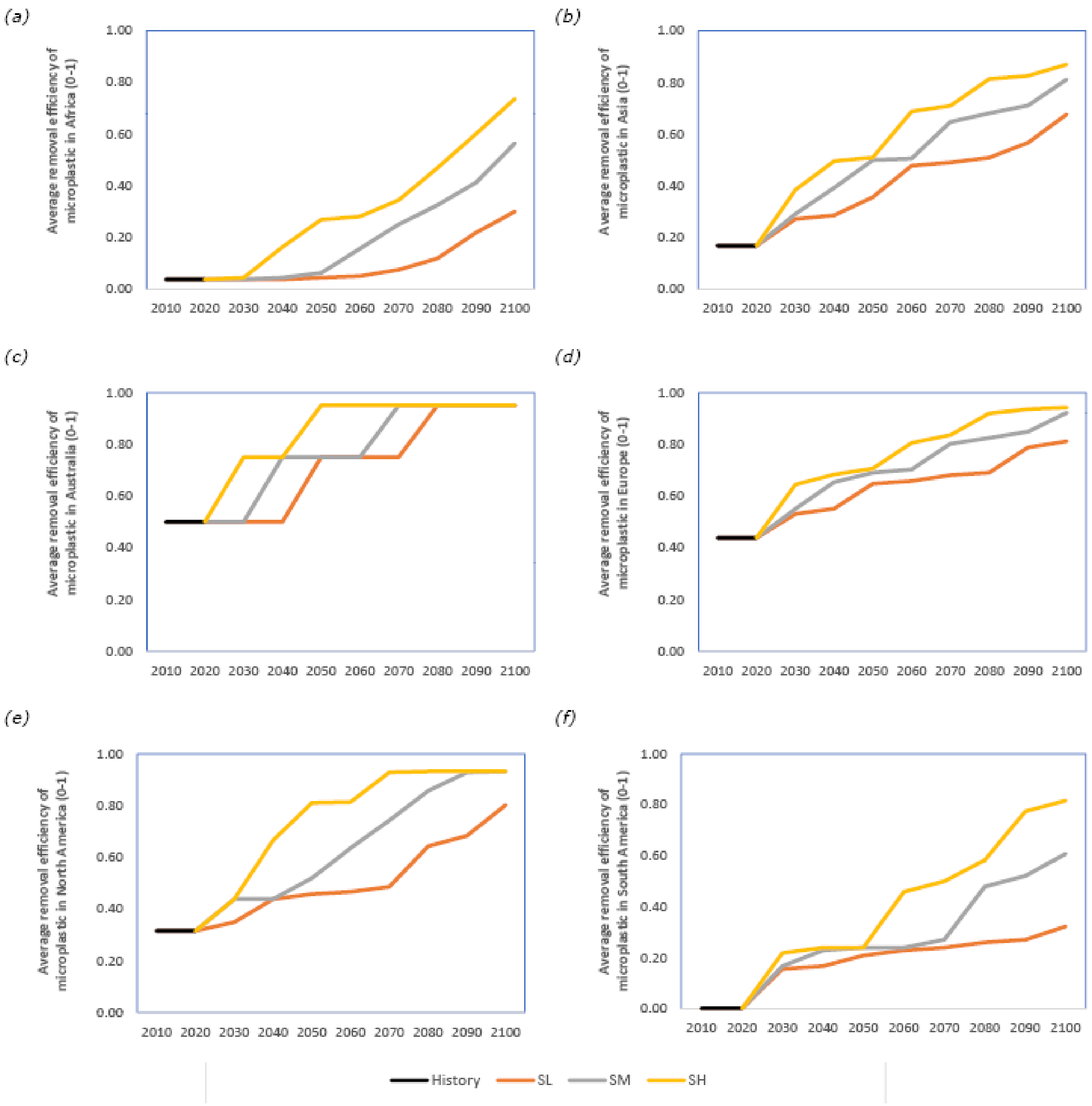

Figure S4. Average removal efficiency of microplastic in (a) African, (b) Asian, (c) Australian, (d) European, (e) North American and (f) South American for the period of 10 years between 2010 and 2100 according to three sanitation scenarios. As the SH^+ scenario has the same population data as the SH scenario, only the SH scenario is shown. Unit is between 0 and 1. The SL, SM and SH represent the Shared Socioeconomic Pathway 2 low, moderate and high scenarios, respectively. Source: See Tables S2-S4 for the model descriptions and the scenario description; van Puijenbroek et al. (2023), van Puijenbroek et al., (2019) and (Strokal et al., 2021).

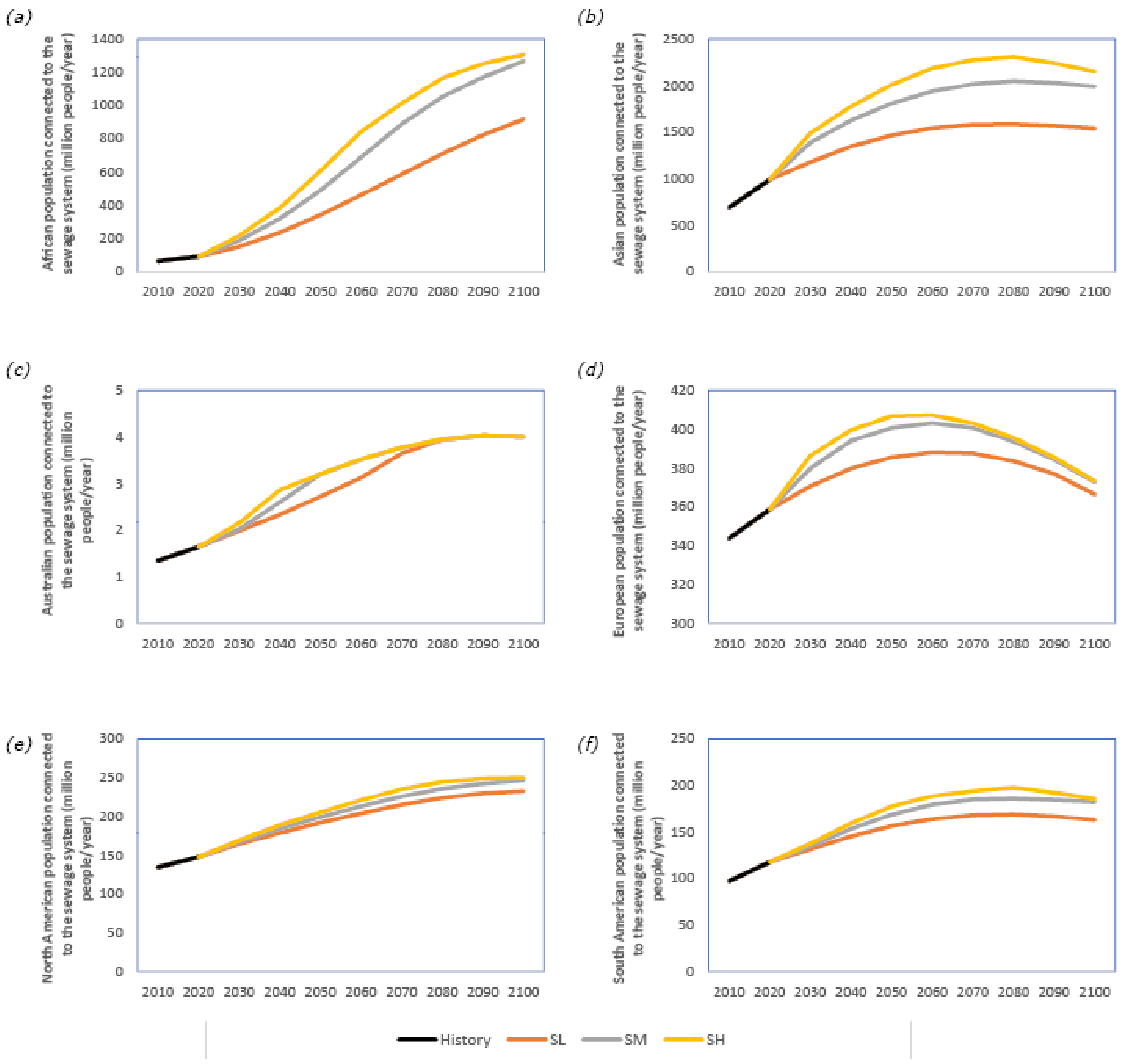

Figure S5. (a) African, (b) Asian, (c) Australian, (d) European, (e) South American and (f) North American population connected to the sewer system for the period of 10 years between 2010 and 2100 according to three sanitation scenarios. As the SH^{+} scenario has the same population data as the SH scenario, only the SH scenario is shown. Unit is million people per year. The SL, SM and SH represent the Shared Socioeconomic Pathway 2 low, moderate and high scenarios, respectively. The total population connected to the sewer systems in sub-basins are summed for each scenario, separately[1]. Source: See Tables S2-S4 for the model descriptions

and the scenario description; van Puijenbroek et al. (2023), van Puijenbroek et al., (2019) and (Strokal et al., 2021).

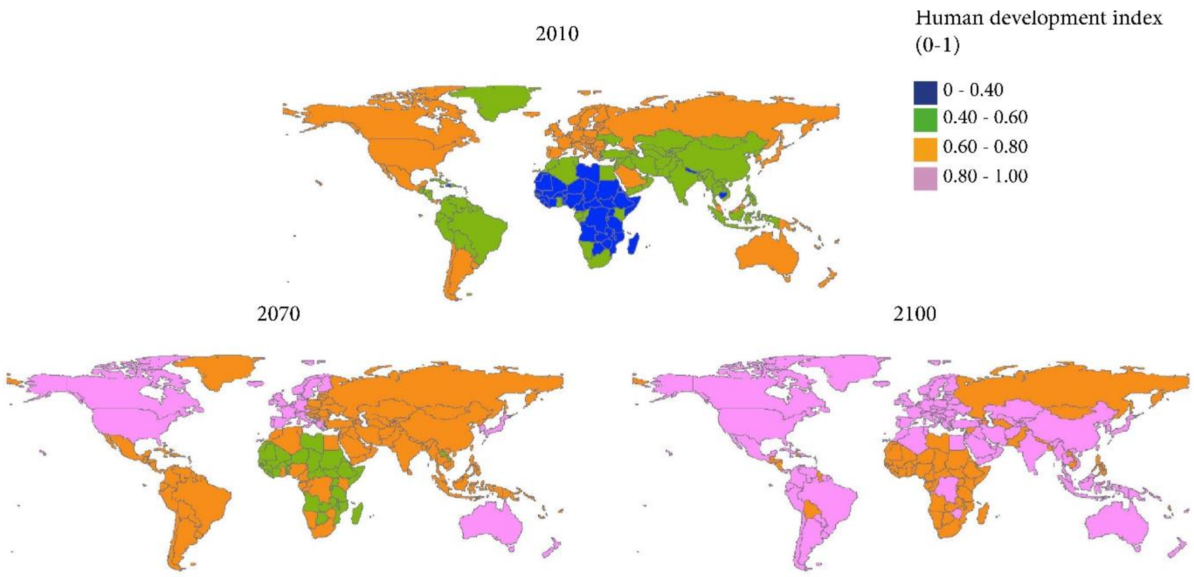

Figure S6. Human Development Index (HDI) for the SM in 2010, 2070 and 2100 at sub-basins. Unit is a percentage[3]. Only the SM scenario is presented since the SL, SM, SH and SH^{+} are used the same HDI values.

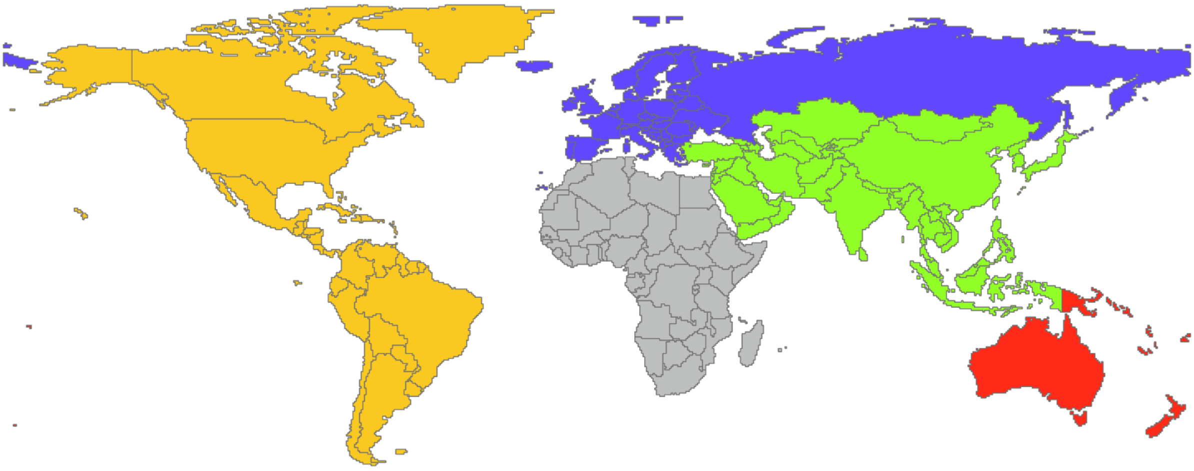

Figure S7. The borders of countries used in this research. Grey, yellow, green, purple and red represents Africa, America, Asia, Europe and Oceania continent, respectively[1].

Table S5. Minimum, average and maximum microplastic inputs in global rivers across four scenarios between 2010 and 2100 (kton km^{-2} $year^{-1}$).

Years	2010			2020			2030			2050			2070			2100		
**Scenarios**	**Min**	**Average**	**Max**	**Min**	**Average**	**Max**	**Min**	**Average**	**Max**	**Min**	**Average**	**Max**	**Min**	**Average**	**Max**	**Min**	**Average**	**Max**
**SL**	6E-07	2E+02	2E+05	3E-04	3E+02	2E+05	8E-07	3E+02	2E+05	1E-06	3E+02	3E+05	1E-06	3E+02	4E+05	2E-06	3E+02	2E+05
**SM**	6E-07	2E+02	2E+05	3E-04	3E+02	2E+05	2E-06	3E+02	3E+05	3E-06	3E+02	3E+05	1E-06	3E+02	4E+05	4E-07	2E+02	1E+05
**SH**	6E-07	2E+02	2E+05	3E-04	3E+02	2E+05	2E-06	3E+02	3E+05	3E-06	3E+02	3E+05	1E-06	2E+02	3E+05	4E-07	1E+02	8E+04
**SH^+**	6E-07	2E+02	2E+05	3E-04	3E+02	2E+05	2E-06	2E+02	3E+05	2E-06	2E+02	2E+05	4E-07	1E+02	1E+05	2E-07	6E+01	4E+04

REFERENCES

(1) Strokal, V.; Kuiper, E. J.; Bak, M. P.; Vriend, P.; Wang, M.; van Wijnen, J.; Strokal, M. Future Microplastics in the Black Sea: River Exports and Reduction Options for Zero Pollution. *Mar Pollut Bull* **2022**, *178*, 113633. https://doi.org/10.1016/j.marpolbul.2022.113633.

(2) van Puijenbroek, P. J. T. M.; Beusen, A. H. W.; Bouwman, A. F.; Ayeri, T.; Strokal, M.; Hofstra, N. Quantifying Future Sanitation Scenarios and Progress towards SDG Targets in the Shared Socioeconomic Pathways. *J Environ Manage* **2023**, *346*. https://doi.org/10.1016/j.jenvman.2023.118921.

(3) Crespo Cuaresma, J.; Lutz, W. The Demography of Human Development and Climate Change Vulnerability: A Projection Exercise. *Vienna Yearb Popul Res* **2016**, *13* (1), 241–262. https://doi.org/10.1553/populationyearbook2015s241.

(4) Jones, B.; O'Neill, B. C. Spatially Explicit Global Population Scenarios Consistent with the Shared Socioeconomic Pathways. *Environmental Research Letters* **2016**, *11* (8), 084003. https://doi.org/10.1088/1748-9326/11/8/084003.

(5) Siegfried, M.; Koelmans, A. A.; Besseling, E.; Kroeze, C. Export of Microplastics from Land to Sea. A Modelling Approach. *Water Res* **2017**, *127*, 249–257. https://doi.org/https://doi.org/10.1016/j.watres.2017.10.011.

(6) Strokal, M.; Spanier, J. E.; Kroeze, C.; Koelmans, A. A.; Flörke, M.; Franssen, W.; Hofstra, N.; Langan, S.; Tang, T.; van Vliet, M. T.; Wada, Y.; Wang, M.; van Wijnen, J.; Williams, R. Global Multi-Pollutant Modelling of Water Quality: Scientific Challenges and Future Directions. *Curr Opin Environ Sustain* **2019**, *36*, 116–125. https://doi.org/10.1016/j.cosust.2018.11.004.

(7) Siegfried, M.; Koelmans, A. A.; Besseling, E.; Kroeze, C. Export of Microplastics from Land to Sea. A Modelling Approach. *Water Res* **2017**, *127*, 249–257. https://doi.org/10.1016/j.watres.2017.10.011.

(8) European Commission. *EU Action Plan: "Towards Zero Pollution for Air, Water and Soil";* Brussels, 2021.

(9) Sun, J.; Dai, X.; Wang, Q.; van Loosdrecht, M. C. M.; Ni, B.-J. Microplastics in Wastewater Treatment Plants: Detection, Occurrence and Removal. *Water Res* **2019**, *152*, 21–37. https://doi.org/10.1016/j.watres.2018.12.050.

(10) van Puijenbroek, P. J. T. M.; Beusen, A. H. W.; Bouwman, A. F. Global Nitrogen and Phosphorus in Urban Waste Water Based on the Shared Socio-Economic Pathways. *J Environ Manage* **2019**, *231*, 446–456. https://doi.org/10.1016/j.jenvman.2018.10.048.

(11) van Wijnen, J.; Ragas, A. M. J.; Kroeze, C. Modelling Global River Export of Microplastics to the Marine Environment: Sources and Future Trends. *Science of The Total Environment* **2019**, *673*, 392–401. https://doi.org/https://doi.org/10.1016/j.scitotenv.2019.04.078.

(12) Strokal, M.; Bai, Z.; Franssen, W.; Hofstra, N.; Koelmans, A. A.; Ludwig, F.; Ma, L.; van Puijenbroek, P.; Spanier, J. E.; Vermeulen, L. C.; van Vliet, M. T. H.; van Wijnen, J.; Kroeze, C. Urbanization: An Increasing Source of Multiple Pollutants to Rivers in the

21st Century. *npj Urban Sustainability* **2021**, *1* (1), 24. https://doi.org/10.1038/s42949-021-00026-w.
